# Moving beyond the Court of Public Opinion: A Citizens’ Jury Exploring the Public’s Values around Funding Decisions for Ultra-Orphan Drugs

**DOI:** 10.3390/ijerph20010633

**Published:** 2022-12-30

**Authors:** Tania Stafinski, Jacqueline Street, Andrea Young, Devidas Menon

**Affiliations:** 1 Health Technology & Policy Unit, School of Public Health, 4-343, Edmonton Clinic Health Academy, Edmonton, AB T6G 1C9, Canada; 2 Australian Centre for Engagement, Evidence and Values, University of Wollongong, Northfields Ave, Wollongong 2522, Australia

**Keywords:** rare diseases, value, citizen jury, resource allocation, drug funding

## Abstract

Health system decision-makers need to understand the value of new technology to make “value for money” decisions. Typically, narrow definitions of value are used. This paper reports on a Canadian Citizens’ Jury which was convened to elicit those aspects of value that are important to the public. The criteria used by the public to determine value included those related to the patient, those directly related to caregivers and those directly created for society. Their choices were not binary (e.g., cost vs. health gained), but rather involved multiple factors (e.g., with respect to patient factors: disease severity, health gained with the drug, existence of alternatives, life expectancy, patient age and affordability). Overall, Jurors prioritized funding treatments for ultra-rare disease populations when the treatment offered significant improvements in health and quality of life, and when the pre-treatment health state was considered extremely poor. The prevalence of the disease by itself was not a factor in the choices. Some of the findings differ from previous work, which use survey methods. In our Citizens’ Jury, Jurors were able to become more familiar with the question at hand and were exposed to a broad and balanced collection of viewpoints before and throughout engaging in the exercises. This deliberative approach allows for a more nuanced approach to understanding value.

## 1. Introduction

Technologic innovation has been identified as a major factor in high and rising health care costs across health systems. In fact, there is evidence that “improved health care technologies generally increase rather than reduce health care expenditures” [1]. This topic of costs has been a “hot-button issue” in Canada for many years now; in particular, the media has been focused on prescription drug costs. More specifically concerning prescription drugs, news sources have been inundated with alarming headlines including “Young Canadians feeling the pinch of sky-high prescription drug prices” [2], “Drug costs rising fast in Canadian health-care spending, report finds” [3] and “Can Canada curb its too-high drug prices?” [4]. Concerns over the high cost of drugs have persisted for decades and policy interventions have been implemented in an attempt to address this. In 1987, the Canadian government established the Patented Medicine Prices Review Board (PMPRB) to help ensure that the prices of patented medicines sold in Canada are not excessive [5]. The pan-Canadian Pharmaceutical Alliance followed twenty-three years later, giving the provinces and territories an opportunity to negotiate jointly for drugs and potentially secure lower prices [6]. In spite of these developments, prices for many new therapies are still seen as excessively high and unaffordable for Canadians; this is true particularly in the area of orphan and ultra-orphan drugs (i.e., drugs that treat rare or ultra-rare conditions).

Due to the generally high prices of these drugs for rare diseases (DRDs), as well as the challenges faced in conducting clinical trials on small patient populations, decision-makers struggle to establish the “value” of these products in order to justify their high price [7,8]. In many existing health funding decision-making processes, the quality-adjusted life year (QALY) is used to measure health outcomes and assess the value of new therapies in economic evaluations [9,10]. However, it has become increasingly recognized that “value” has many dimensions, which are not all adequately captured through clinical outcomes and the QALY, as currently used, may not capture all of the relevant outcomes or benefits. These include the value to family and caregivers as well as to society overall. Recently, a proposal was made to amend PMPRB’s regulatory framework to include new price regulatory factors that will better enable the PMPRB to consider the price of patented medicines in relation to their value to patients and impact on the health care system [5]. Originally intended to come into effect on 1 January 2022, the changes have now been delayed until further notice.

How is this broader perspective of value of orphan drugs to be captured? Demands for public engagement have been increasing based on the argument that the public best represents Canadian “social values” [11]. This is seen as particularly important in publicly funded health systems, where the public comprises the taxpayers. Studies eliciting the opinion of the public have been published, but these were survey-based [12,13,14,15]. The findings from these studies raised concerns about including public perspectives where these might be influenced by variable knowledge of rare diseases, framing of the survey questions, and the construction of the provided trade-off scenarios [11,16]. To effectively include public values and to elicit the trade-offs the public might be willing to make, we need informed deliberative and balanced engagement with a diverse group of people who hold, and are willing to promote, the interests of the public. 

In this study, we aimed to elicit public values and the trade-offs the public is willing to make through the use of a deliberative method of engagement known as a Citizens’ Jury. The Jury was recruited to reflect the perspectives that the public in Alberta, Canada, would bring to the development of criteria for decision-making in public funding for ultra-rare disease treatments. We asked a descriptively representative group of the Alberta public the question: what criteria should be used in decision-making to fund high-cost therapies for ultra-rare conditions?

## 2. Materials & Methods

In representative democracies such as Canada, decisions on which new health technologies to fund and for whom are made by individuals elected based on political platforms that reflect only a handful of the issues they face once in power. Therefore, in some circumstances, they may seek to understand the public’s sentiment in order to formulate policies that are consistent with social values. Those circumstances often relate to healthcare and access to new services that promise significant health gains but at a high cost. Thus, they are often politically charged and receive considerable media attention. Deliberative democratic methods offer an approach to eliciting the views of the public around such issues [17]. They allow people to consider policy issues in depth and in relation to the potential consequences to others rather than only from an individual viewpoint. In addition, they support discussion of policy topics according to set rules which ensure the quality of debate and its legitimacy as a public engagement exercise. One of these methods is Citizens’ Juries. Citizens’ Juries are conducted based on the premise that a small diverse group drawn from a population and presented with relevant evidence can deliberate in a way that reflects the values and interests of the general public [18]. Unlike other methods of public engagement (e.g., surveys), this process aims to combine information, time, scrutiny, deliberation, independence, and authority [19]. To date, it has been used to understand the public’s views on a variety of health topics [20,21,22,23,24,25,26,27]. 

A jury is typically comprised of 12–25 members of the public who as a group are descriptively representative of the community [19]. The jury deliberates on a question or charge over 2–4 days. Expert witnesses from a broad range of perspectives relevant to the topic are make presentations and answer questions. Subsequently, the jurors engage in deliberations to reach common ground or consensus [19]. 

One issue that continues to challenge health systems and, in turn, decision-makers around the world relates to reimbursement for ultra-orphan drugs [28], These drugs target conditions that affect populations too small for the kinds of clinical trials typically required for regulatory and reimbursement approval. Thus, there is significant uncertainty around their effectiveness. At the same time, they often comprise the first disease-modifying treatment available and, as such, promise to significantly impact the health and well-being of individuals whose options have traditionally been limited to supportive care [11,29].

Often discussion around coverage for these treatments focus on whether rare diseases are somehow “special” [30]. However, there are many criteria that need to be considered simultaneously and the decision cannot be a binary one [31]. Therefore, a deliberative approach in the form of a Citizens’ Jury, which allows for trade-offs to be considered deliberatively, was selected as the method of choice for this study.

The formation and conduct of the Jury established for this study are described in detail below.

### 2.1. Formation of the Citizens’ Jury 

Potential jurors were recruited via letter mail from Central and Northern Alberta [20]. Information packages were sent to 3000 names and addresses obtained through random sampling of a commercial database owned by Canada’s national postal service (Canada Post). The letters explained the nature and purpose of a Citizens’ Jury and this particular Jury, logistical details and potential risks and benefits of participation. Respondents consented to a follow-up phone interview in which eligibility was assessed. To ensure participation regardless of financial circumstances, respondents were offered a CAD 400 honorarium. Jurors travelling from outside the Greater Edmonton Area were provided with accommodation while those from Edmonton, but without a vehicle, were offered transportation support.

Brief follow-up interviews determined respondents’ sociodemographic characteristics (age, ethnicity, employment status, and household income), employment in a health-related field and affiliations with special interest groups such as patient organizations (see Supplementary Materials S1 for the patient Information Letter). Those meeting either of the latter two criteria were excluded to ensure the Jury was comprised of ‘ordinary citizens’ whose voices might otherwise not be heard. Eligible respondents were grouped by gender and age and then stratified by education level and household income. Jurors were purposively selected to match Alberta’s population distribution. Random sampling was used to select a juror when several respondents with the same set of characteristics were identified.

### 2.2. Conduct of the Citizens’ Jury

The Jury was held at a hotel conference centre in Edmonton, Alberta over two and a half days. It included presentations by experts, scenario-based decision-making exercises and small and large group deliberation. A team of seven male and female researchers facilitated the exercises. All researchers were, at a minimum, Masters prepared, had experience in facilitating focus groups and had conducted work in the rare diseases area.

On day one (half-day), jurors were briefed about the jury process and participated in an ‘icebreaker’ exercise. They completed a questionnaire assessing their views (pre- and post-jury) on the importance of different criteria related to health funding decision-making (see Supplementary Materials S2 for the questionnaire and a detailed description of the exercises). In addition to rating and ranking four decision-making criteria (number of patients affected, initial health state, prognosis without treatment, and expected health gain), Jurors were required to make funding choices between treatments in a variety of patient populations. 

On days one and two, the jurors heard from experts on the role of priority setting in health care, challenges related to rare diseases and access to orphan drugs, and the impact of rare diseases on patients’ and carers’ lives. Witnesses included: former decision-makers from the provincial and regional health authority level, a physician who treats rare disease patients, a pharmaceutical company representative, a patient, and the parent of a patient. On Day 1, the experts participated in a panel session, reviewing five different technology profiles and choosing two to ‘fund’ (Table 1a). The experts were asked to explain their rationale, giving the Jurors an opportunity to understand the different decision-making criteria considered by the experts. Throughout the jury process, Jurors had opportunities to ask questions of the witnesses and interrogate the evidence. 

Over the course of the Jury, the Jurors engaged in a range of exercises designed to elicit the values they held and the way in which they might trade off those values in making decisions about public funding of health technologies. (See Table 2 for a summary of the exercises). The first of these required a similar exercise to the experts namely selecting five technologies—from a list of ten—which they considered should be funded and their rationale for their selection. (See Table 1b for the list of ten technologies).

### 2.3. Analysis and Reporting of Jury Findings

Individual Juror responses and group consensus were documented for each exercise. Facilitators reviewed their notes with the Jurors at the end of each Exercise. The deliberations were audio-recorded and transcribed. Two researchers analyzed the documented responses and transcripts thematically using some pre-established codes and identifying other codes as they emerged (Supplementary Materials S3). Themes capturing the criteria that Jurors considered most important to funding decision-making were identified through constant comparative analysis.

This manuscript was written following the COnsolidated criteria for REporting Qualitative research (COREQ) checklist [32], (Supplementary Materials S4) and citizens’ jury checklist.

## 3. Results

### 3.1. Citizens’ Jury

One hundred and twenty-three people responded to the letter mail (4.1%). All respondents were interviewed via telephone by two researchers. The sociodemographic profile of the sixteen Jurors selected to participate in the Jury can be found in Table 3. There were no dropouts during the study.

### 3.2. Which Technologies Should We Fund and Why?

Facilitators documented the technologies selected by each group member in Exercise 1 using flip charts, noting the number of Jurors who “voted” for each one, after which the groups began to deliberate. From their deliberations, the high-priority decision-making criteria were identified across all three groups. These are described in Table 4. After being questioned by the expert witnesses the following day and reflecting on their choices most jurors continued to support their choices and refer to the same criteria they had identified previously.

One group changed their minds choosing to fund IPT for juvenile diabetes instead of the shingles vaccine. They provided a rationale related to the age of the patients citing that juvenile diabetes affects children “*for their lifetime*“ vs. the “*short term impact*” of the disease treated by the technology that they chose to give up (shingles vaccine). Additionally, the jurors referred to the significant caregiver burden stating that funding would allow caregivers to return to the workforce (“*that means there’s productivity increase based on the parents being freed up and… joining the work force again*”).

### 3.3. Trading Benefits and Harms

Jurors from each group attempted to establish a “point system” to facilitate their decisions based on the size of the impact of the technology but quickly abandoned it, making decisions that were not always for the highest point option, but instead reflected their individual values. As the complexity in the trade-off exercises increased, Jurors re-considered using a point system but did not adhere to it (“*going with the gut feeling, really, on a few of them*”). Jurors often stated that their choice was for the option with ‘more gain’ (“*most change on this one*”; “*there’s a greater change overall*”), but this did not always correspond to the highest point option, suggesting that certain criteria were considered more important. 

Across all three groups, maximizing the benefits experienced by patients was considered a priority (“*for me, it’s patient benefit first*”). Jurors sacrificed benefits for both caregivers (“*…as a caregiver, you suck it up if it’s going to make the patient better*”) and/or society (“*…when I’m picking the large patient benefit over the large society benefit, I’m thinking someone’s life and the value compared to basically money*”) to increase the benefits obtained by patients. The Jurors even sacrificed benefits for caregivers and/or society that were greater in magnitude than the benefits obtained by patients. Some Jurors suggested that, over time, benefits gained by patients would have a trickle-down effect on caregivers and society that the exercise did not reflect (“*I would think that when you’ve got…a large patient benefit, it should essentially ripple down. The caregiver benefits increase, your societal benefit should increase*”).

Decisions to trade-off benefits between caregivers and society generated greater uncertainty and disagreement, where responses to trade-offs typically resulted in “majority-rules” decisions and consensus could not be reached. Some Jurors prioritized caregiver benefits next after patient benefits (“*caregiver would be my second [priority], because I’m thinking caregivers are family members*”) while other considered them to be the lowest priority (“*if I had to sacrifice the caregiver for society, I would do that*”; “*bear in mind that society is the one that’s paying for everything, so you can’t discount them entirely*”). 

### 3.4. Valuing Health Gains

An increased life expectancy was highly valued as long as the Jurors were ‘okay’ with the patients’ status in other criteria. If patients would live longer but in extreme pain or with severe physical dysfunction, Jurors were less comfortable funding their treatment (“*you’re just extending their pain. I don’t want to be in extreme pain for five years*”; “*I would not want to be responsible for extending that suffering*”). While they did consider the possibility that increasing life expectancy may mean patients are alive to benefit from future innovations, this was not deemed a valuable enough gain to warrant funding an option (“*do you buy somebody more time and suffering on the off chance that something could be discovered in 10, 15 years?*”). Not all Jurors were comfortable making this decision on behalf of patients, however (“*we can’t make decisions for these people when we have the chance to prolong their lives*”). Jurors were also reluctant to fund treatments that provided significant benefit in function, pain, or depression, if the patients’ life expectancy remained at less than a year. Jurors were particularly concerned with leaving patients in significant pain (“*my thing that got me was the extreme pain*”). Independence was also a significant consideration and the Jurors leaned towards funding options in which it was implied that the patients would become more independent and have less reliance on caregivers (“*you’re able to carry out daily life*”).

### 3.5. Valuing the Opportunity Cost

Jurors also considered the health state of the patients whom they did not fund, suggesting that disease severity affected their decisions. If one option provided greater benefit, but the other option treated a ‘sicker’ population, Jurors would sometimes choose the latter, considering it “*the least that they could do*” (“*then obviously I will always… as long as there’s some [“severe”] in some of those conditions*”). 

Where the trade offs were simple (Exercise 1) Jurors often chose to fund technologies affecting a larger number of people. However, as the complexity increased (Exercise 3) Jurors were prepared to fund treatment for 100 patients over 10,000 patients with another condition provided that they considered the gains significant enough. Jurors did not make these decisions lightly. They acknowledged the implications of having 10,000 untreated patients, and thus needed to be ‘okay’ with the state that the 10,000 would remain in before they would choose to treat the 100.

### 3.6. Trading Small Health Gains 

Jurors demonstrated a limited willingness to pay for ‘small gains’. They were prepared to ‘fund’ new therapies in six of the eight scenarios presented but typically only if the price of the new treatment was less than double the price of an existing therapy. When the price increased past this point, they worried that funding the new therapy would cut the number of patients who could be treated in half (“*[for that price] it’s two patients getting this treatment in the old way*”). For five of the ‘funded’ therapies, Jurors were willing to pay between 25% and 75% more than the cost of the existing treatment. Jurors recognized the value of convenience and the burden of out-of-pocket costs (“*if you’re living outside of town and you have to come in, then that’s an expense that a person has to bear*”), but saw a limit to how much these gains were worth (“*and with the knowledge of how much that cost me, I still say between [dollar amount range] would be my max*”). For the sixth therapy, Jurors were willing to pay more than double the cost of the existing therapy. In this scenario, the new treatment provided patients with a somewhat greater life expectancy and a better quality of life during their final months. In this scenario, the Jurors placed significant value on increased life expectancy when quality of life was also improved and/or considered acceptable. 

There were two scenarios where Jurors were unwilling to pay any additional money for a new treatment. In the first scenario, the existing treatment involved patients taking 10 pills, six times a day, while the new treatment only involved 1 pill, six times a day. The Jurors felt that “*for a pill that has the exact same effect, [they] just [couldn’t] justify spending more money…*” In the second scenario, Jurors were asked to fund a test that identifies patients with a genetic mutation guaranteeing they will develop a particular disease later in life, but no treatment is provided until they become symptomatic. Jurors wondered why the “*[patients] can’t pay on an individual basis*”, stating that the “*[price] is a small number that could be paid for by the individual*”.

### 3.7. Reliability and Validity of Exercises

In Exercises 2 and 3, Jurors in all three groups responded identically to the questions repeated within and across the groups, demonstrating that the exercises were both reliable and valid.

### 3.8. Pre- and Post-Jury Questionnaire

The majority of Jurors ranked all four criteria (‘number of patients affected’, ‘initial health state’, ‘prognosis without treatment’, and ‘expected health gain’) between moderately and very important (most leaning towards important or very important). The preferences of most of the jurors were unchanged or only slightly changed by participation in the jury. The exception was ‘initial health state’. Most Jurors placed a higher importance on this post-jury compared with pre-jury.

Changes were noted in the ranking of all four decision-criteria. Pre-Jury, the majority of Jurors ranked ‘number of patients affected’ as the most important criteria but as the least important post-Jury. The average rankings of ‘prognosis without treatment’ and ‘expected health gain’ were lower post-Jury. The average ranking of ‘initial health state’ increased. 

Finally, Jurors demonstrated a greater willingness to fund small patient populations post-Jury. In scenarios where the small patient population were either more severely ill or benefited from a greater health gain with treatment than the larger population, the majority of Jurors chose to fund the small population. In some scenarios post-Jury, Jurors were also willing to treat populations with a lower ‘expected health gain’ but who were considered more severely ill. This is consistent with Part A and B of the survey, which demonstrated that the Jurors increased the value they placed on ‘initial health state’ over the course of the weekend.

## 4. Discussion

A 2015 review reported on 62 distinct deliberative consultations around health-related matters [31]. Community or citizens juries were used in 20 of these. Typically, juries were convened to address questions dealing with justice and fairness in policy making. In the area of ultra-orphan drugs, justice and fairness are often key aspects of decision-making.

To our knowledge currently there are no other published studies using a Citizens’ Jury to explore the Canadian public’s willingness to fund drugs for ultra-rare conditions. However, researchers have reported using surveys to collect similar information [33,34] One national online survey reported public priorities for ‘effect on quality and quantity of life’ and ‘severity of the disease’, as well as limited value being place on rarity alone [33]. A second national online survey reported ‘improved quality of life’ and ‘effective health care’ as top priorities for consideration in funding decision-making [35]. In this survey half of the participants were willing to fund drugs for rare diseases regardless of whether or not they were found to be cost-effective. The findings of these surveys are similar to those of this study, which found that Jurors prioritized funding treatments for ultra-rare disease populations when the treatment offered significant improvements in health and quality of life, and when the pre-treatment health state was considered extremely poor. In contrast to findings of the Jury, the first survey reported that the public was unwilling to accept the opportunity cost for funding an orphan drug if it was at the expense of a common condition [33]. Given the change in attitude seen in our study, following engagement with the complexity of scenarios and deliberation with others, this is not surprising. Research has suggested that study participants may be more averse to funding orphan drugs due to low levels of public awareness around orphan drug issues and when survey questions present decisions as a zero—sum game, (i.e., decisions to fund orphan drugs directly result in reductions in funding for other drugs without exploring consequences). 

The effectiveness of deliberative methods, including citizens’ juries, has been studied in a 5-arm randomized trial in the US [35]. It concluded that deliberation increased knowledge and changed attitudes, particularly on the use of evidence. It also concluded that public deliberation may be especially helpful for those issues in health where “members of the public have limited knowledge (or even incorrect information) about a technically complex topic yet hold strong beliefs and attitudes” [35]. 

The specific approach used in this project has undergone methodological inquiry in a previous study. Deliberation among the jury resulted in jurors wanting to know more about a topic and react to what they heard from others—therefore, they became more informed on the topic. It also showed that there were changes in jurors’ attitudes as deliberation took place. Finally, it was shown that 2 separate juries who underwent the same deliberative exercises on the same project reached the same conclusions. They also retained their views months after the jury had been held [21].

This jury has resulted in 3 sets of criteria which are considered important while considering funding ultra-orphan drugs: patient-related criteria, caregiver-related criteria and societal criteria. This reflects the complexity of the decision problem, which is whether to fund a specific drug or not, and the broad view on what “value” means. These criteria need to be considered simultaneously and it must be recognized that they will be weighted differently, according to the specific circumstances. Clearly, funding decisions on new ultra-orphan drugs cannot be made in a “formulaic” manner. This may challenge decision-makers who might prefer a single measure (e.g., cost/QALY) toe represent “value”. However, the realities about ultra-orphan drugs are complex and different approaches are needed. 

### Limitations

It is possible that members of the public who respond to recruitment letters are different from those who do not (e.g., members of the public with special interest in certain health areas). To avoid this selection bias, the screening questionnaire included two questions to exclude anyone who may already be actively engaged in the health care system or patient organizations.

## 5. Conclusions

This Citizens’ Jury demonstrates that an informed Albertan public given time to deliberate with others does not prioritize prevalence alone when making funding decisions on ultra-orphan drugs. The Jurors indicated that they also valued initial and final health states and amount of health gain. Additionally, they were willing to fund ultra-orphan drugs over common conditions when the small population gains significant benefit with treatment and the large population is believed to be ‘okay’ going without. The findings of this Jury are timely as Canada moves forward in its efforts to better assess the value of high-cost therapies during reimbursement decision-making.

## Figures and Tables

**Table 1:** Technology profiles used for Expert Witness panels and Exercise 1.

Technology	Indication
a. For expert witness panels	
Everolimus Eliglustat Smart-e-pants Robotic assisted surgery Hepatitis C screening	Tuberous Sclerosis Complex * Gaucher Disease * Prevention of pressure ulcers in people with mobility issues Prostate cancer Screening program to identify Hepatitis C positive Albertans
b. For Exercise 1	
Insulin Pump Therapy	Type 1 diabetes
Zostavax^®^ Vaccine	Shingles
Prophylactic breast and ovary removal	Women with BRCA1/2 gene mutations
Anaplastic Lymphoma Kinase (ALK) screening	Test for ALK mutation in non-small cell lung cancer patients to determine if they will respond to Crizotinib *
Ivacaftor	Cystic Fibrosis patients with specific gene mutation *
Stem cell transplantation	Heart failure
Monoclonal antibody treatment	High cholesterol
Glycerol phenylbutyrate	Urea cycle disorder *
Gene therapy	Choroideremia *

* Rare indication.

**Table 2:** Jury deliberation exercises to demonstrate held values and indicated trade-offs for publicly acceptable decision-making.

Exercise (Day)	Rationale for Exercise	Materials/Information Provided	Structure	Exercise
1 (Days 1 & 2)	Explore criteria the public thinks should be considered in funding decisions and what impact prevalence of the disease has on their views	Description of 10 different technologies to review (see Table 1 and Supplementary Materials S2).	Deliberation in small and large group information given one day in advance	Jurors were asked to select five technologies from their list of ten (Table 1) to ‘fund’ and to explain their rationale.
2 (Day 2)	Explore public values around treatment benefits offered to society compared to individuals or caregivers	Set of 29 simple trade-off scenarios describing two treatment options by the magnitude of benefit to patients, caregivers, and society (small, medium, or large) (Supplementary Materials S2)	Deliberation in small and large group. To test exercise reliability and validity, two scenarios were repeated in each small group and four scenarios across all small groups	Jurors were asked to review the scenarios individually and select one option to fund. The groups then deliberated to reach for consensus on each scenario.
3 (Day 3)	Explore public understandings of how policy-makers should deal with public ‘backlash’ to decisions	Jurors told of public “backlash” to their decision to not fund a technology in Exercise 1. An expert witness (acting as a Deputy Minister of Health or patient organization representative or pharmaceutical company representative) advocated to each group (the ‘decision-making committee’) attempting to ‘change their decision’.	Deliberation in small groups.	Groups were asked to deliberate. If the groups did reconsider and decide to fund the technology, they were required to ‘give up’ funding one of the five they had originally selected.
4 (Day 3)	Explore public values with respect to how the public weighs disease prevalence against other factors when considering health gain	Jurors were presented with 30 different scenarios comparing two treatment options. For each option, a patient populations’ current health state and their expected health gains with treatment were described in terms of life expectancy, physical functioning, pain and discomfort, cognitive functioning, and depression and anxiety.	Deliberation in small groups followed by report-back session. To test exercise reliability and validity, two scenarios were repeated in each small group and four scenarios across all small groups	Jurors were asked to ‘fund’ one treatment based on this information, individually and as a group. Once consensus was reached, Jurors were asked if they would change their decision knowing that the patient population for the option they funded was only 100 patients vs. 10,000 patients in the option they chose not to fund.
5 (Day 3)	Examining the extent to which the public values marginal benefits (i.e., small health gains) over other factors when considering health gain	Jurors were presented with eight scenarios describing an existing treatment for a condition as well as a new treatment that is the same price and offers a benefit over the existing therapy that could be considered “small” (e.g., convenience) (Supplementary Materials S2).	Deliberation as a jury	Jurors were asked if they would continue to fund the new treatment over the existing treatment at increasing prices to identify the point at which they would no longer fund the new treatment.

**Table 3:** Socio-demographic profile of the Jury.

Characteristic	Number of Jurors (%)
**Gender**	
Male	8 (50)
Female	8 (50)
**Age**	
18–24	2 (12.5)
25–34	2 (12.5)
35–44	2 (12.5)
45–54	3 (18.75)
55–64	3 (18.75)
65–74	2 (12.5)
>74	2 (12.5)
**Education (highest level)**	
<High school	1 (6.25)
High school	5 (31.25)
Post-secondary diploma	5 (31.25)
Undergraduate degree	3 (18.75)
Graduate degree	2 (12.5)
**Annual household income (CAD, before taxes)**	
<CAD 25,000	2 (12.5)
CAD 25,000–CAD 45,000	4 (25)
CAD 46,000–CAD 70,000	4 (25)
CAD 71,000–CAD 100,000	3 (18.75)
>CAD 100,000	3 (18.75)
**Employment status**	
Employed	9 (56.25)
Unemployed	3 (18.75)
Retired	4 (25)
**Ethnicity**	
Asian	1 (6.25)
Caucasian	14 (87.5)
Metis	1 (6.25)
**Geographic location**	
Urban	13 (81.25)
Rural	3 (18.75)

**Table 4:** Criteria jurors used to justify choices to fund or not fund technologies (Exercise 1).

Criteria	Rationale	Examples/Quotes
Criteria related to patients		
Disease severity	Jurors considered the “*[severity] of the disease and how the treatment relieves [symptoms]*”, prioritizing technologies for conditions that they considered to be more severe	Shingles vaccine “*they [symptoms] can get bad for some people, like, extremely severe*”). Some jurors rejected funding for technologies which did “*not [treat illnesses] that will necessarily kill [the patients]*”
Amount of ‘gain’	Decisions were based on Jurors’ judgements of the amount of benefit that the patients would gain from the treatment and how this would affect their quality of life.	Important benefits were patients’ Independence—“*ability to be independent or not independent*” Convenience—“*ease of use*” Exposure to stigma—“*lessen[ing] a possible stigma*” Jurors valued technologies that they saw as providing “*more permanent solutions*”—cures rather than ongoing treatment
Alternatives	Jurors prioritized technologies for which there were no alternative treatments or for which they considered the alternative undesirable or less effective Some technologies were specifically not selected for funding because an alternative was available, even if it had disadvantages	Undesirable alternative:, e.g., stem cell therapy for heart failure—“*it’s an alternative to surgery so you don’t have to go through all of that*” Less effective:, e.g., monoclonal antibody therapy for high cholesterol—“*people that are on statins maybe don’t benefit as much…this would fix that*” Available alternative:, e.g., Glycerol phenylbutyrate for urea cycle disorder—”*it’s already got a solution*”; “*there’s a way to treat the disease that has a drawback*”. Potential alternative through life style change:, e.g., rejecting funding for monoclonal antibody therapy for high cholesterol because patients could engage a “*change in lifestyle…eating healthier…*”
Life expectancy	Jurors took life expectancy with and without treatment into consideration when making their decisions and excluded those treatments which did not change life expectancy or those conditions which already had a long life expectancy.	Jurors excluded: Technologies which provided diagnostic information but which did not extend life—e.g., ALK screening because it did not “*do anything for your death clock*” They also excluded those conditions where life expectancy was considered long without the new technology, e.g., Ivacaftor for Cystic Fibrosis—“*it said even without treatment you live past 50…so you still have a pretty good life expectancy even without Ivacaftor*”
Age	Some Jurors felt that the average age of the patient population is important to consider when making funding decisions. However, this sentiment was not unanimously shared.	Suicide prevention in teenagers—“*points for [that] it was for the youth…you know, the people of tomorrow*” Some jurors saw children as *a* “*priority because you’re talking about small children going to school, growing, and being able to participate more fully…*”
Affordability	Some jurors rejected public funding for technologies on the basis of patient-level affordability, suggesting that the funding should be diverted to more expensive and therefore unaffordable technologies.	Shingles vaccine—“*not something that would be too heavy on a senior to pay on a one-time basis…*” and “*extended family could assist with [payment]*”.
Criteria directly related to caregivers:		
Caregiver burden	Jurors recognized that these diseases affect “*more than just the patients*” and wondered, “*if [patients] are now dependent on someone else, what is [their] caregiver’s quality of life?*” Consequently, they prioritized technologies that reduced caregiver burden and increased caregiver quality of life.	Insulin pump for children—“*it is better for the parents…they don’t have to worry about their kids*”).
Criteria directly related to society		
Prevalence	Jurors selected technologies treating diseases with a higher prevalence because they provide “*the biggest bang for the buck*” and “*help the majority of people*”.	In a few cases, Jurors explicitly chose not to fund technologies that affected a smaller number of patients (e.g., Ivacaftor)—“*it improves their quality of life, but it’s such a small number [of patients]*”).
Societal burden	Jurors prioritized treatments that would allow patients to “*be less of a burden on the health care system*” in the future and allow their family caregivers to “*get back into the work force*”	Example of reducing burden for patients: Prophylactic breast and ovary removal in BRCA1/2 positive patients). Example of reducing burden for carers: IPT for Juvenile diabetes).
Innovation	Jurors prioritized technologies that they saw as innovative and that may have “*applications in other areas*” including to permit removal of less effective treatments	E.g., innovative gene therapy—“*it will have the potential affect of [treatments] for other disease if it’s effective…*”). They also saw the use of new, “*more perfected and more potent*” therapies as allowing for the discontinuation of less effective treatments, freeing up resources for researchers and “*the professionals… the nurses, the health care aides*”, etc. to “*focus on the needs of other diseases.*”

## Supplementary Materials

The following supporting information can be downloaded at: https://www.mdpi.com/article/10.3390/ijerph20010633/s1, Supplementary Materials S1: Information Letter. Supplementary Materials S2:Pre- and post-questionnaire. Supplementary Materials S3: Sample coding tree Supplementary Materials S4: COREQ checklist.
