# Supplementary material for: Moving beyond the Court of Public Opinion: A Citizens’ Jury Exploring the Public’s Values around Funding Decisions for Ultra-Orphan Drugs
Source: Int J Environ Res Public Health. 2022 Dec 30;20(1):633. doi: 10.3390/ijerph20010633 (PMC9819519; doi:10.3390/ijerph20010633)
Supplement: Supplementary file 1 [file ijerph-20-00633-s001.zip › Supplementary Materials S1.pdf]

Supplementary Materials S1

Recruitment letter mail

UNIVERSITY OF ALBERTA

School of Public Health
Department of Public Health Sciences
[redacted]

[Insert potential participant's name]
[Insert address]
[Insert city]
[Insert postal code]

POTENTIAL JUROR INFORMATION LETTER

Title of Project: Determining social values in health care

Principal Investigator:
[redacted] University of Alberta, School of Public Health [redacted]

Co-Investigator:
[redacted] University of Alberta, School of Public Health [redacted]

[Insert date]

Dear [Insert potential participant's name]:

You are being asked to participate in a telephone survey. This survey will be used to select 32 Albertans from in or around Central and Northern Alberta (from Red Deer northward) to take part in a project that will look at how ordinary citizens could help governments understand the value of new health technologies.

New health technologies need to be carefully examined before the government of Alberta or Alberta Health Services can decide whether or not they should be paid for and used to treat patients. Because there are so many new technologies, it is not possible to fund all of them. Someone has to choose which ones are the most important. Until now, the public has not been involved in making these decisions.

The purpose of this project is to try out a new way to involve the public in health policy decisions. It is called a Citizens' Jury.

UNIVERSITY OF ALBERTA

School of Public Health
Department of Public Health Sciences
[redacted]

What is a Citizens' Jury?
A Citizens' Jury is made up of 12 to 16 people from the general public who have been brought together to consider a topic or issue and give an informed opinion on it.

What happens during a Citizens' Jury?
For 2-3 days, the jury learns some of the background to the topic. It listens to witnesses who provide information and talk about different things the jury might want to consider. Witnesses can be doctors, researchers, health care managers, people from government or the regional health authority and patients. The jury gets to ask witnesses questions and then discuss what they have heard among themselves. At the end, it comes up with conclusions or recommendations on how the issue should be tackled. The findings are written in a report that is given to the people who asked for the jury to be held in the first place. Before they get the report, jurors are sent copies and asked to make any comments. Jurors typically receive copies within one week of the jury session and are asked to provide comments within 2 weeks. It should take about one hour to review.

The sessions are recorded. The tapes will be used to make sure that the written report of the session is accurate.

How long will it take?
The Jury is being held on different dates at the [redacted]. If you respond and would like to participate, you may be selected to participate on one of these Juries.

The Jury will meet in Edmonton for two and a half days, from [redacted]. The session will begin with an introductory dinner at **5:30 pm on April 17th**. On the **following two days**, the sessions will start at **9:30 am and last until 5:00 pm**.

Will you be paid for this?
You will be paid $400.00 for the two and a half days. Your travel costs will also be paid. If you come from outside the Edmonton area and need to drive for more than one hour to get to the place where the jury will be held, we will pay for your hotel. If you need to hire a babysitter in order to come to this, we will pay for that too. We will also provide coffee breaks and lunches while you are attending the sessions.

What are the benefits and risks of being in this study?
You will be helping decision-makers in the province determine which new health technologies to assess for public funding. Governments cannot afford to look at all technologies, and they need to make tough choices over what technologies are more important than others. Some of the information you may hear on how governments decide might make you feel uncomfortable. You

[redacted]

information you may hear on how governments decide might make you feel uncomfortable. You are free to withdraw from the study at any time if you wish. You do not have to give a reason. Also, you have the right to not answer any question that you do not want to answer.

What about confidentiality?
The meeting will not be open to the public, and the names of jurors and witnesses will not appear in the final report. Those who agree to take part in the jury will be asked to keep the names of jurors and witnesses private. However, we cannot guarantee that other jury members will maintain the confidentiality of what is discussed. You do not need to use your real name when the jury is being held.

All information will be held confidential (or private), except when professional codes of ethics or legislation (or the law) require reporting. The information you provide will be kept in a secure area (i.e., locked filing cabinet) for a minimum of 5 years. Your name or any other identifying information will not be attached to the information you gave. Your name will also never be used in any presentations or publications of the project's results. The information gathered for this project may be looked at again in the future to help us answer other questions. If so, the ethics board will first review the project to ensure the information is used ethically.

Are you interested in being considered for the Jury?
We are sending this letter to 3,000 individuals in or around the Edmonton area. From the people who respond and wish to participate, a maximum of 16 individuals will be chosen per Jury (32 individuals, total). To make sure that the Jury can represent the views of the entire community, we need to have a mix of people from different age groups, parts of the region, and backgrounds. Therefore, we will not be able to accept all individuals who wish to participate.

To be considered for the jury, you will need to complete a survey that asks a few questions about you. The survey will be done over the telephone and should last no more than ten minutes. The interviewer will call you. The answers you provide will be used to help us get a mix of people for the jury. A confidential identification number will be assigned to you so that your name will never appear on the survey. That way, the researcher involved in selecting jurors will not know the names of people who have agreed to participate.

If you wish to participate in the jury and are willing to complete the telephone survey, please complete the attached consent form and return it by **April 1, 2015**. Letters may be returned via fax to [redacted] or scanned and returned via email to [redacted]. Please note that if you decide not to participate, there will be no negative consequences to yourself.

The principal researcher for this project is [redacted] Public Health Sciences, University of Alberta. Mailing address [redacted]. Telephone: [redacted]; Fax: [redacted]; e-mail: [redacted].

[redacted]

Should you need additional information on this project, please contact [redacted] at [redacted]. Should you have any questions about your rights as a study participant, please contact the University of Alberta Health Research Ethics Board at [redacted].

Your consent

Your signature on the attached consent form indicates that you understand the information about your participation in the project and that you agree to participate in the telephone screening survey. Please keep these pages for future reference.

Sincerely,
[redacted]
Professor, Health Policy and Management

[redacted]

TELEPHONE SURVEY CONSENT FORM

Title of Project: Determining social values in health care

Principal Investigator:
[redacted]
University of Alberta
Department of Public Health Sciences [redacted]

Co-Investigator:
[redacted]
University of Alberta
Department of Public Health Sciences [redacted]

I have read the information letter and agree to take part in the telephone survey, which will be used to select participants for the jury:

Signature of participant: ______________________ Printed name:________________________

Date: ________________ (day/month/year)

Telephone number: ____________________ (home) ______________________ (work)

Email address: ________________________

Preferred time during which you would like to be contacted for the telephone survey: *(please write preferred time of day (morning, afternoon, or evening))*

Screening questionnaire

[redacted]

SCREENING QUESTIONNAIRE

ID Number: ____________________

Name: ________________________

Interviewer: ___________________

Date of interview:_______________

Telephone number:______________

Interviewer: My name is "----" and I am calling to follow-up on a consent form we received indicating that you would like to be considered for the Citizens' Jury on social values for healthcare. The jury will be asked to come up with ideas about what should be taken into account when governments are trying to determine the value of a new health technology and whether it should be funded when there isn't enough money to fund everything. First, I would like to thank you for your interest in the project and ask you if you have any questions so far? Please feel free to stop me at any time if you are unclear about something or would like to ask a question.

Interviewer: As explained in the information letter, we are trying to select 16 jurors who represent Albertans. This means that we need a mix of males and females, and people from different age groups, income levels, educational backgrounds, and professions. We also need people who are unlikely to voice their views otherwise. Your answers to the questions that I will be asking you will help to make sure that we get things right! Please be aware that you do not have to answer any questions that you do not want to and you may stop the interview at any time. If it's alright with you, I would like to begin now.

Questions:

1. First of all, are you still willing and able to participate in the jury, which will take place at the [redacted]?

☐ Yes
☐ No

If yes, proceed to question 2. If no, stop the interview.

[redacted]

2. Please state your address (including postal code):____________________

3. How long have you lived in Alberta?

4. Are you male or female?________________

5. Please tell me which one of the following age categories you fall into? (check off one of the boxes)

☐ 18 – 24
☐ 25 – 34
☐ 35 – 44
☐ 45 – 54
☐ 55 – 64
☐ 65 – 74
☐ >74

6. In what country were you born?

7. How many years have you lived in Canada?

8. How would you describe your ancestry?
If interviewee asks what you mean, explain that Stats Canada uses the following categories: First Nations or Aboriginals, South Asian, Chinese, Black, Filipino, Latin American, Arab, Southeast Asian, West Asian, Korean, Japanese, or visible minority not included elsewhere)

8. What is the highest level of education you have completed? (check off one of the following boxes)

☐ Less than high school
☐ High school
☐ Post-secondary diploma
☐ University degree
☐ Post graduate degree
☐ Other (please specify__________________________)

[redacted]

9. Are you currently working for pay or for profit? Yes / No

If yes, go to question 10. If no, skip to question 13.

10. Which of the following best describes your work status? (check off one or more of the boxes)

- ☐ Full-time
- ☐ Part-time
- ☐ Seasonal
- ☐ Self-employed
- ☐ On contract
- ☐ In a salaried position

11. To which of the following household income categories do you belong, before taxes and deductions: (check off one of the boxes)

- ☐ < $25,000
- ☐ $25,000 to $45,000
- ☐ $46,000 to $70,000
- ☐ $71,000 to $100,000
- ☐ > $100,000

12. Have you ever been employed by the health care system (e.g., health care professional, administrator, support staff, etc.)? Yes / No

If yes, go to question 13. If no, skip to question 15.

13. In what capacity? (e.g., nurse, pharmacist, doctor, respiratory technologist, etc.)

14. For how long?

15. Does any immediate member of your family currently work in health care? Yes / No

If yes, go to question 16. If no, skip to question 17.

16. Who of your immediate family works in health care and in what capacity?

[redacted]

17. Have you ever been associated with or a member of any patient group or society? Yes / No

18. Have you ever worked for a medical product or private health care company? Yes / No

If yes, go to question 19. If no, skip to question 19.

18. In what capacity and do you still work for one?

Thank you for taking the time to answer these questions. When we have finished all of our interviews and selected the 16 jurors, we will send you a letter stating whether or not we were able to include you in the jury. We plan to have these letters sent out within the next two weeks. In the meantime, if you have any questions or concerns, please feel free to contact me.
