# Supplementary material for: Moving beyond the Court of Public Opinion: A Citizens’ Jury Exploring the Public’s Values around Funding Decisions for Ultra-Orphan Drugs
Source: Int J Environ Res Public Health. 2022 Dec 30;20(1):633. doi: 10.3390/ijerph20010633 (PMC9819519; doi:10.3390/ijerph20010633)
Supplement: Supplementary file 1 [file ijerph-20-00633-s001.zip › Supplementary Materials S2.pdf]

Supplementary Materials S2

Pre- and post-Jury Questionnaire

CITIZENS JURY QUESTIONNAIRE

Please imagine that you are the provincial Minister of Health. Your job is to decide which new health services the province should pay for. These health services may help different groups of patients, and there isn't enough money to fund all of them. When deciding which ones to fund, you might consider:

**Number of patients affected**	How many patients might benefit from the health service?
**Current health**	How ill are the patients in the group who might benefit from the health service?
**Life expectancy without treatment**	What is the life expectancy of patients in the group if the service is not funded?
**Health gain**	How much will the health of patients in the group improve if the service is funded?

PART A

Please answer the following questions by placing a check mark in the box that best describes your views:

1. How important is it to think about the number of patients who could benefit when deciding which new health services to fund?

Very important ☐
Important ☐
Moderately important ☐
Of little importance ☐
Not important at all ☐

2. How important is it to think about how ill patients currently are when deciding which new health services to fund?

Very important ☐
Important ☐
Moderately important ☐
Of little importance ☐
Not important at all ☐

3. How important is it to think about what might happen to patients if the new health service is not funded when deciding which new services to fund?

Very important ☐
Important ☐
Moderately important ☐
Of little importance ☐
Not important at all ☐

4. How important is it to think about how much the health of patients might improve if they get the new health service when deciding which new services to fund?

Very important ☐
Important ☐
Moderately important ☐
Of little importance ☐
Not important at all ☐

PART B

Please rank the following four factors that might be used to help determine the priority of different patient groups for health care in order of importance to you, starting with the most important one:

Number of patients
Current health
Prognosis without treatment
Health gain

Most important

1.____________________________________

2.____________________________________

3.____________________________________

4.____________________________________

Least important

PART C

Please imagine that you only have enough money to fund one of two treatments. Treatment A is used to treat illness A (patient group A), while treatment B is used to treat illness B (patient group B). The two groups of patients are different.

The table on the next page shows the possible characteristics of the patient groups.

**Characteristic**	**Categories**	**Description**
Number of patients	Few	Less than 100
	Many	More than 10,000
Current health	Severely ill	Unable to perform daily activities (working, family or leisure); in extreme pain or discomfort; depressed
	Moderately ill	Unable to perform some daily activities; in moderate pain; mildly depressed
	Mildly ill	Able to carry out daily activities; in mild pain
Life expectancy without treatment*<br>*Some disease progress slowly and others progress very quickly	Less than 1 year	
	1 to 5 years	
	More than 5 years	
Health gain with treatment	Maximum	Health returns to normal (what it was before the illness); life expectancy returns to normal (what is was before the illness)
	Moderate	Health does not return to normal (pre-illness), but patients are able to perform most daily activities; life expectancy doubles from what it was without treatment
	Minimal	Health does not return to normal and patients are not able to carry out most daily activities on their own; life expectancy doubles from what it was without treatment

Questions 1 through 18 ask you to make a choice between funding treatment for Group A or Group B. Alternatively, you can choose to fund both groups equally or indicate that you would prefer that someone else makes the choice. For each question, please place a check mark in the box that best describes your view.

QUESTION 1

	GROUP A	GROUP B
**Number of patients**	Few	Few
**Current health**	Moderately ill	Mildly ill
**Life expectancy without treatment**	Less than 1 year	1 to 5 years
**Health gain with treatment**	Maximum	Moderate

Please check one of the following boxes:

Fund Group A ☐

Fund Group B ☐

Divide funds equally between the groups ☐

Let someone else decide ☐

QUESTION 2

	GROUP A	GROUP B
**Number of patients**	Many	Few
**Current health**	Mildly ill	Mildly ill
**Life expectancy without treatment**	Less than 1 year	1 to 5 years
**Health gain with treatment**	Moderate	Maximum

Please check one of the following boxes:

Fund Group A ☐

Fund Group B ☐

Divide funds equally between the groups ☐

Let someone else decide ☐

QUESTION 3

	GROUP A	GROUP B
**Number of patients**	Many	Many
**Current health**	Severely ill	Moderately ill
**Life expectancy without treatment**	Less than 1 year	More than 5 years
**Health gain with treatment**	Minimal	Moderate

Please check one of the following boxes:

Fund Group A ☐

Fund Group B ☐

Divide funds equally between the groups ☐

Let someone else decide ☐

QUESTION 4

	GROUP A	GROUP B
**Number of patients**	Few	Few
**Current health**	Severely ill	Mildly ill
**Life expectancy without treatment**	A few weeks	1 to 5 years
**Health gain with treatment**	Maximum	Maximum

Please check one of the following boxes:

Fund Group A ☐

Fund Group B ☐

Divide funds equally between the groups ☐

Let someone else decide ☐

QUESTION 5

	GROUP A	GROUP B
**Number of patients**	Few	Many
**Current health**	Moderately ill	Mildly ill
**Life expectancy without treatment**	More than 5 years	1 to 5 years
**Health gain with treatment**	Maximum	Maximum

Please check one of the following boxes:

Fund Group A ☐

Fund Group B ☐

Divide funds equally between the groups ☐

Let someone else decide ☐

QUESTION 6

	GROUP A	GROUP B
**Number of patients**	Few	Many
**Current health**	Severely ill	Moderately ill
**Life expectancy without treatment**	Less than 1 year	Less than 1 year
**Health gain with treatment**	Minimal	Maximum

Please check one of the following boxes:

Fund Group A ☐

Fund Group B ☐

Divide funds equally between the groups ☐

Let someone else decide ☐

QUESTION 7

	GROUP A	GROUP B
**Number of patients**	Many	Few
**Current health**	Moderately ill	Moderately ill
**Life expectancy without treatment**	More than 5 years	Less than 1 year
**Health gain with treatment**	Moderate	Moderate

Please check one of the following boxes:

Fund Group A ☐

Fund Group B ☐

Divide funds equally between the groups ☐

Let someone else decide ☐

QUESTION 8

	GROUP A	GROUP B
**Number of patients**	Few	Few
**Current health**	Moderately ill	Mildly ill
**Life expectancy without treatment**	1 to 5 years	More than 5 years
**Health gain with treatment**	Minimal	Moderate

Please check one of the following boxes:

Fund Group A ☐

Fund Group B ☐

Divide funds equally between the groups ☐

Let someone else decide ☐

QUESTION 9

	GROUP A	GROUP B
**Number of patients**	Many	Many
**Current health**	Moderately ill	Severely ill
**Life expectancy without treatment**	Less than 1 year	1 to 5 years
**Health gain with treatment**	Maximum	Moderate

Please check one of the following boxes:

Fund Group A ☐

Fund Group B ☐

Divide funds equally between the groups ☐

Let someone else decide ☐

QUESTION 10

	GROUP A	GROUP B
**Number of patients**	Few	Few
**Current health**	Moderately ill	Mildly ill
**Life expectancy without treatment**	Less than 1 year	1 to 5 years
**Health gain with treatment**	Minimal	Moderate

Please check one of the following boxes:

Fund Group A ☐

Fund Group B ☐

Divide funds equally between the groups ☐

Let someone else decide ☐

QUESTION 11

	GROUP A	GROUP B
**Number of patients**	Many	Many
**Current health**	Severely ill	Mildly ill
**Life expectancy without treatment**	Less than 1 year	1 to 5 years
**Health gain with treatment**	Moderate	Maximum

Please check one of the following boxes:

Fund Group A ☐

Fund Group B ☐

Divide funds equally between the groups ☐

Let someone else decide ☐

QUESTION 12

	GROUP A	GROUP B
**Number of patients**	Few	Few
**Current health**	Moderately ill	Mildly ill
**Life expectancy without treatment**	1 to 5 years	More than 5 years
**Health gain with treatment**	Moderate	Maximum

Please check one of the following boxes:

Fund Group A ☐

Fund Group B ☐

Divide funds equally between the groups ☐

Let someone else decide ☐

QUESTION 13

	GROUP A	GROUP B
**Number of patients**	Few	Many
**Current health**	Mildly ill	Moderately ill
**Life expectancy without treatment**	1 to 5 years	More than 5 years
**Health gain with treatment**	Moderate	Minimal

Please check one of the following boxes:

- Fund Group A ☐
- Fund Group B ☐
- Divide funds equally between the groups ☐
- Let someone else decide ☐

QUESTION 14

	GROUP A	GROUP B
**Number of patients**	Few	Few
**Current health**	Severely ill	Severely ill
**Life expectancy without treatment**	Less than 1 year	1 to 5 years
**Health gain with treatment**	Minimal	Moderate

Please check one of the following boxes:

- Fund Group A ☐
- Fund Group B ☐
- Divide funds equally between the groups ☐
- Let someone else decide ☐

QUESTION 15

	GROUP A	GROUP B
**Number of patients**	Few	Few
**Current health**	Severely ill	Mildly ill
**Life expectancy without treatment**	More than 5 years	More than 5 years
**Health gain with treatment**	Moderate	Maximum

Please check one of the following boxes:

- Fund Group A ☐
- Fund Group B ☐
- Divide funds equally between the groups ☐
- Let someone else decide ☐

QUESTION 16

	GROUP A	GROUP B
**Number of patients**	Many	Many
**Current health**	Moderately ill	Severely ill
**Life expectancy without treatment**	Less than 1 year	More than 5 years
**Health gain with treatment**	Minimal	Minimal

Please check one of the following boxes:

- Fund Group A ☐
- Fund Group B ☐
- Divide funds equally between the groups ☐
- Let someone else decide ☐

QUESTION 17

	GROUP A	GROUP B
**Number of patients**	Many	Many
**Current health**	Severely ill	Severely ill
**Life expectancy without treatment**	More than 5 years	1 to 5 years
**Health gain with treatment**	Moderate	Maximum

Please check one of the following boxes:

Fund Group A ☐

Fund Group B ☐

Divide funds equally between the groups ☐

Let someone else decide ☐

QUESTION 18

	GROUP A	GROUP B
**Number of patients**	Few	Many
**Current health**	Mildly ill	Moderately ill
**Life expectancy without treatment**	1 to 5 years	More than 5 years
**Health gain with treatment**	Moderate	Minimal

Please check one of the following boxes:

Fund Group A ☐

Fund Group B ☐

Divide funds equally between the groups ☐

Let someone else decide ☐

Exercise 1

Technology profiles for expert witness panel

Name of technology: Everolimus for Tuberous Sclerosis Complex

What is it?
Everolimus is a drug taken by mouth every day that helps to shrink non-cancerous tumours that can grow in the brain, kidneys, lungs, and skin of patients with Tuberous Sclerosis Complex (TSC). The tumours are called subependymal giant cell tumours (SEGA). TSC is a rare genetic disorder. Mutations in two genes cause cells to divide in an uncontrolled way, leading to the development of tumours in multiple organs in the body. Everolimus works by stopping a particular protein that triggers cells to grow and divide from working properly. In this way, it is able to slow down or stop the tumours from continuing to grow. Patients treated with Everolimus take this drug for the rest of their lives.

What might it replace?
Depending on the location of tumours, they may be surgically removed. However, while the tumours are slow growing and non-cancerous, they can come back after surgery. Tumours on the skin may be treated with lasers. Tumours in the brain can block the flow of fluids around the brain, causing a build-up of pressure. The tumours can also cause seizures, developmental delays, and behavioural problems. Anti-epileptic medications may help to control the seizures.

Who might benefit?
TSC affects 1 in 6,000 births. Approximately 3,500 Canadians have TSC. Patients whose tumours cannot be removed through surgery could benefit from Everolimus. Patients who continue to develop tumours despite surgery could also benefit from Everolimus.

How might they benefit?
Although the tumours are slow growing, they can affect how organs function and cause sudden death. By shrinking or stopping the growth of these tumours, further damage to organs may be prevented and seizures may be reduced. The risk of developing fluid on the brain may be reduced. About 25% of patients on Everolimus experience fever and acne. About 80% develop small sores in the mouth or on the lips, similar to canker sores.

What happens if it isn't funded?
Patients will continue to receive treatment to manage symptoms, such as surgery and anti-epileptic drugs.

Name of technology: Eliglustat for Gaucher Disease

What is it?
Eliglustat is a drug taken by mouth every day that replaces an enzyme lacking in patients with Gaucher Disease. Therefore, it is called an enzyme replacement therapy (ERT). It is not a cure, so patients need to take the drug for the rest of their lives in order to continue to receive benefit from the treatment.

Gaucher Disease is caused by a genetic mutation received from both parents. The mutation prevents the body from making enough of a specific enzyme needed to break down or digest a fatty substance, called a lipid, produced when red blood cells are recycled by the body. As a result, cells fill up with undigested lipid. These cells are called Gaucher cells. They build up in many places in the body, but most commonly the liver, spleen and bone marrow, where it interferes with normal functions, such as the production of platelets and red blood cells. This results in bleeding, bruising, anemia, and massive enlargement of the spleen and liver. The lipid also triggers the loss of minerals in the bones and reduced blood flow, causing significant pain and weakening of the bones.

There are three types of Gaucher disease. The most common form is Type 1. The different types are based on whether there is neurological involvement. Patients with Type 1 have no neurological involvement. Since Eliglustat cannot cross the blood brain barrier, it is used for patients with Type 1 Gaucher disease.

What might it replace?
Currently, patients with Type 1 Gaucher Disease are treated with enzyme replacement therapy that is given intravenously over 1 to 2 hours every 2 weeks. Like the oral therapy, it is not a cure, so patients need to continue to receive the infusions in order to experience benefit. The treatment has been shown to relieve and, in some cases, reverse the symptoms of the disease. Less than 15% of patients experience side effects to the treatment, all of which are temporary. Many are infusion reactions, which can be controlled or prevented by administering medications before the infusion.

Who might benefit?
Type 1 Gaucher Disease affects 1 in every 100,000 individuals in Canada. Patients who are currently receiving intravenous ERT may benefit from Eliglustat.

How might they benefit?
Since Eliglustat is taken by mouth, patients do not need to go for infusions every 2 weeks. Common side effects include stomach discomfort, bloating, and pain in the arms and legs, which can be managed without medical attention.

What happens if it isn't funded?
Patients will continue to receive ERT intravenously.

Name of technology: Smart-e-pants - Special therapeutic underwear for prevention of pressure ulcers

What is it?
Smart-e-Pants are a new tool to prevent pressure ulcers (also called pressure sores or bedsores). These undergarments, which look like bicycle shorts, contain electrodes that painlessly stimulate the nerves and gluteal muscles (in the buttocks), increasing circulation at the base of the spine. In this way, the pants mimic the movements of able-bodied people whose tissues receive much-needed oxygen and blood flow.

Pressure ulcers can be superficial skin injuries, but often they are serious, deep tissue wounds that are painful, difficult to heal, and increase the risk for further adverse events, such as infection.

What might it replace?
Current ways to prevent pressure ulcers include regular turning of patients by nursing staff and other caregivers, training patients to practice movement exercises to relieve pressure at regular intervals, and the use of special wheelchair cushions, mattress covers and beds. Preventing pressure ulcers requires the use of many different interventions but, overall, preventive measures are less costly than the costs of treating pressure ulcers.

Who might benefit?
Patients with mobility issues, such as those confined to wheelchairs due to spinal cord injuries or confined to beds at home or in long-term care facilities could benefit from Smart-e-Pants. About 86,000 Canadians have spinal cord injuries. Over their lifetime, most (86%) will develop pressure ulcers. In addition, over 200,000 Canadians live in nursing homes or long-term care facilities. Many of these individuals are bed-ridden or in wheelchairs for much of their day. Pressure ulcers can develop in a matter of days, or even hours, in individuals who are immobile, and particularly in those with fragile skin due to age, poor circulation or atrophied muscles.

How might they benefit?
Preventing pressure ulcers increases individuals' quality of life, and decreases pain, as well as the need for intensive outpatient wound care or hospitalization. Smart-e-Pants are worn for about 12 hours per day. The electrical stimulation improves muscle tone and tissue circulation in the buttocks area – the area most susceptible to pressure ulcers. Because the pants may reduce the need for frequent turning and repositioning by others, patients may feel more independent, improving emotional well-being.

What happens if it isn't funded?
Despite the use of other best practice interventions, many patients still develop pressure ulcers which put them at risk for further health problems and the likelihood they will require more care.

Name of technology: Robotic surgery for prostate cancer

What is it?
Robotic surgery or robot-assisted surgery uses robotic devices to help surgeons perform complex procedures, such as prostate cancer surgery, with greater precision, flexibility and control. This allows for more precise removal of the cancer while minimizing surgical nerve damage that may cause permanent urinary incontinence or impotence.

Prostate cancer is usually affects older men (over the age 60). Often, it is a slow growing cancer that does not cause symptoms, so monitoring, rather than active treatment, may be an option for some men. However, some prostate cancers are more aggressive and are likely to spread beyond the prostate gland if not removed. Also, as the cancer grows within the prostate, it blocks the genitourinary tract, causing problems with urination and defecation. Surgical removal of the prostate gland and some of the surrounding tissue (radical prostatectomy) is the main treatment option.

What might it replace? Other options
Currently, the prostate gland is removed through conventional surgical techniques called a radical prostatectomy. With conventional techniques, 50% to 90% of patients experience permanent side effects, including incontinence and impotence.

Who might benefit?
Prostate cancer is the most common cancer in men. Each year, over 23,000 men in Canada are diagnosed with prostate cancer, and approximately 4,000 will die from this disease. For many of these men, the prostate cancer will not cause symptoms, and they will eventually die from other causes. However, in Canada, about 8,000 men each year will need prostate surgery for cancer treatment.

How might they benefit?
Preservation of the nerves surrounding the prostate is important to ensure normal urinary tract function and to avoid causing impotence. Less invasive surgery using robotic guidance may also mean less risk of surgical or wound infection, a shorter hospital stay, and faster patient recovery.

What happens if it isn't funded?
If robotic surgery is not funded, patients will continue to undergo radical prostatectomy. Some patients may refuse this treatment, knowing that it may cause permanent side effects (incontinence and impotence), which can significantly affect their quality of life.

Name of technology: Hepatitis C Screening

What is it?
Hepatitis C screening involves a blood test for specific antibodies to the hepatitis C virus and can detect the disease in patients without symptoms.

Hepatitis C is an infectious disease that primarily affects the liver. It is spread through contact with infected blood. Risk factors for hepatitis C include sharing needles for injected drug use with an infected person; having sexual intercourse with an infected person; being pricked by a needle with infected blood on it (which can happen in certain occupations, such as nursing); getting a tattoo, piercing, manicure, pedicure, or other medical or dental procedure with contaminated equipment; being born to an infected mother; or having had a blood transfusion or organ transplant prior to 1992 in Canada.

Many people infected with hepatitis C do not know they have the disease because they do not have any symptoms or they experience symptoms many years after infection. The hepatitis C virus can be spread to others during this time. Often by the time that symptoms of hepatitis occur, the liver is already severely damaged.

What might it replace?
Currently, there is no hepatitis C screening program in Alberta.

Who might benefit?
It has been estimated that there are currently about 250,000 people in Canada with hepatitis C. However, there may be many more, since most people have no symptoms.

How might they benefit?
Hepatitis C screening could lead to an early diagnosis of the disease, which may prevent liver scarring, liver failure, and liver cancer. Thus, patients could experience improvements in quality of life and survival. It could also prevent the spread of hepatitis C within the community.

What happens if it isn't funded?
If hepatitis C screening isn't funded, many people living with the hepatitis C virus will remain undiagnosed until they experience symptoms. They will continue to infect other individuals.

Technology profiles for Jurors

Technology name: Insulin Pump Therapy for Type 1 Diabetes

What is it?
Insulin pump therapy (IPT) is a way of controlling blood sugar in patients with Type 1 Diabetes. Type 1 Diabetes is an autoimmune disease where the pancreas does not produce insulin, a hormone that controls the level of glucose (sugar) in your blood. Insulin pumps are small automated devices that deliver insulin to the body through a plastic tube. The tubing is inserted into the skin using a small needle and taped in place and the pump is worn outside the body (e.g., in your pocket). Insulin pumps mimic the natural release of insulin by the pancreas through two insulin delivery systems: 1) a steady system programmed to deliver a small amount of insulin continuously between meals and during sleep, and 2) a bolus system programmed to deliver a larger amount of insulin before snacks and meals or to correct a high blood glucose level.

What might it replace?
The standard treatment for Type 1 Diabetes is multiple dose insulin injections, which involve using a needle and syringe or insulin pen to inject a mixture of different insulin types throughout the day and night.

Who might benefit?
Type 1 Diabetes is usually diagnosed in childhood or adolescence. It accounts for about 5% of all Diabetes and is estimated to affect nearly 100,000 Canadians. Insulin pump therapy is not suitable for all patients with Type 1 Diabetes but can be particularly useful for young children, patients with a lifestyle that makes multiple insulin injections difficult, such as competitive athletes, or patients who are not able to control their blood glucose using multiple insulin injections.

How might they benefit?
Insulin pump therapy is a predictable and precise method of delivering insulin to the body without having to perform multiple insulin injections throughout the day and night. As a result, patients on insulin pump therapy may experience less pain and discomfort, less embarrassment, and improved ability to function physically (less episodes of high and low blood glucose, improved sleep and energy). Caregivers of patients using insulin pump therapy may also experience less stress and anxiety (less time worrying about giving insulin injections and more time for themselves and other family activities), improved ability to function physically (better sleep and increased energy) and better predictability for daily planning. Some parents of young children are able to return to work, since they don't need to be there to inject multiple doses of insulin.

What happens if it isn't funded?
Without insulin pump therapy, patients with Type 1 Diabetes will continue to manage high blood glucose with multiple dose insulin injections.

Technology name: Zostavax® Vaccine for Shingles

What is it?
Zostavax® is a vaccine that can protect men and women 50 years of age and older from shingles. Shingles is a disease caused by a reactivation of the virus that causes chickenpox. The Zostavax® vaccine contains a weakened version of the chickenpox virus which, once injected into the body, boosts the immune system to help prevent shingles. Zostavax® does not guarantee that individuals will not contract shingles, but it can reduce the duration and severity of the disease and the chances of experiencing complications associated with the disease.

What might it replace?
There is no alternative shingles vaccine. Varivax®, a routine childhood vaccine used to prevent chickenpox, has been shown to reduce the chances of contracting shingles. Similar to Zostavax®, Varivax® can reduce the severity of the disease and the chances of experiencing complications associated with the disease.

Who might benefit?
About 130,000 Canadians are diagnosed with shingles each year. About 1 in 3 Canadians will get shingles in their lifetime. It typically occurs in older adults and immunocompromised individuals.

How might they benefit?
Shingles often presents as a rash, typically on the face or torso (the main part of the body to which the neck and limbs are attached). The rash eventually blisters, scabs and may cause scarring. Nerve pain is the most common complication of shingles, and it may last for several months after the rash is gone. Zostavax® can reduce the pain and disfigurement (rash) associated with shingles and improve their ability to function physically (better mobility and sleep). Caregivers of people who receive Zostavax® may experience less stress and anxiety, since it can be emotionally difficult to take care of someone who is experiencing pain and discomfort. Zostavax® contributes to the innovation of new prevention strategies for shingles and an understanding of the nature of the virus in relation to the chickenpox virus. Also, the vaccine may reduce health care costs associated with treating shingles.

What happens if it isn't funded?
Currently, there is no treatment for shingles. Patients with shingles use antiviral and pain medications to reduce the pain and duration of shingles. If Zostavax® isn't funded, people will have a higher chance of getting shingles. If patients do get shingles, they will continue to manage it with antiviral and pain medications. Children will continue to receive the chickenpox vaccine to reduce their chances of contracting chickenpox and shingles.

Technology name: Preventive breast and ovary removal for women with a rare gene that predisposes them to breast and ovarian cancer

What is it?
Prophylactic surgical removal of both breasts (bilateral mastectomy) and ovaries (oophorectomy) is performed to reduce the risk of developing breast or ovarian cancer in women who have a family history that increases their risk of developing these diseases.

What might it replace?
Currently, genetic testing to identify women at risk, and regular monitoring to ensure early detection of cancers, such as with annual mammograms, physical examination, and blood tests, are the main options for these women.

Who might benefit?
Each year, about 24,000 women in Canada are diagnosed with breast cancer and about 5,000 women will die from the disease. Similarly, 2,700 Canadian women are diagnosed with ovarian cancer each year, and approximately 1,750 will die from this disease. An estimated 5% to 10% of these women carry a genetic mutation (BRCA1 and / or BRCA2) that puts them at a much higher risk for both breast and ovarian cancer. The prevalence of the BRCA1/2 mutations is estimated to be from one in 500 to one in 1,000 Canadian women. Current guidelines recommend that women with a family history of breast or ovarian cancer receive genetic testing.

How might they benefit?
In women with the BRCA1/2 mutations, preventive removal of the breasts and ovaries substantially reduces the risk of developing these cancers (reduced to about 5% from 40% to 90%). For many, particularly those who have had their children, preventive surgery is appealing because decreasing their risk of developing these cancers reduces anxiety and improves their quality of life – despite the surgical risks involved.

What happens if it isn't funded?
Genetic testing to identify women at risk and regular monitoring to ensure early detection of cancers will continue to be provided.

Technology name: Anaplastic Lymphoma Kinase (ALK)) screening

What is it?
ALK screening is a test used to screen tissue samples from tumours in patients with non-small cell lung cancer (NSCLC) for a particular mutation called the ALK mutation. Only patients with this mutation have been shown to respond to a chemotherapy called crizotinib. While crizotinib is not curative, patients who receive this chemotherapy experience less pain and coughing.

What might it replace?
ALK screening would not replace any one specific type of cancer screening method.

Who might benefit?
The ALK mutation is found in 2% to 7% of NSCLCs, which represents 1, 462 individuals per year in Canada. ALK positive patients have the worst outcomes of all lung cancer patients, with an average overall survival of approximately 1 year.

How might they benefit?
Patients whose tumours have the ALK mutation would receive crizotinib, a targeted chemotherapy. ALK positive patients on crizotinib gain 6 months during which the cancer doesn't progress, compared to 3 months on standard treatment. They also have a better quality of life, experiencing less coughing and pain. While their chances of living past one year are only marginally better than with standard treatment, this additional time may be give them the opportunity to get their affairs in order.

What happens if it isn't funded?
Patients will continue to be treated with general, non-targeted chemotherapies, but may not live as long as they would have otherwise and may have a poorer quality of life.

Technology name: Ivacaftor for Cystic Fibrosis

What is it?
Ivacaftor is a drug that helps patients with Cystic Fibrosis (CF) who have a specific mutation in one of their genes. This mutation produces a faulty protein that does not function properly. Ivacaftor helps restore the function of the protein, allowing for a proper flow of salt and fluids on the surface of the lungs. This helps to thin and loosen the think, sticky mucous that builds up in the lungs of CF patients.

Patients with CF experience symptoms such as persistent cough with thick mucus, wheezing and shortness of breath, metabolism problems, and frequent chest infections (which can eventually become life-threatening). Most patients do not live beyond 50 years of age.

What might it replace?
Current treatment for CF patients involves managing the symptoms of the disease, or problems that occur as a result of the symptoms, such as infections. Ivacaftor does not replace current treatment. It is taken in addition to current treatment.

Who might benefit?
Only CF patients with the specific mutation will benefit from Ivacafor. There are approximately 115 such patients in Canada.

How might they benefit?
Patients taking Ivacaftor are able to gain weight and their lung function and overall quality of life improves. Because Ivacaftor loosens the mucus in the lungs and vital organs, it relieves some symptoms, such as shortness of breath, and decreases the chances of getting a chest infection, which could mean fewer hospital visits. Ivacaftor may also provide long term protection against certain organ degeneration commonly seen in CF patients and prevent CF-related diabetes.

What happens if it isn't funded?
Patients will continue to receive treatment for symptoms only.

Technology name: Stem cell transplantation for heart failure

What is it?
Stem cells are a particular kind of cell that can produce more cells of the same or of a different cell type. Stem cells can be produced by the body (for example, in the bone marrow) to regenerate tissue in response to injury. They can also come from sources such as excess embryos after in-vitro fertilization procedures, or from umbilical cords discarded after childbirth. In patients with heart failure, stem cells are injected into the heart to help it to repair the damage and regenerate new tissue.

Heart failure is damage to the heart that prevents it from pumping enough blood to meet the body's needs for oxygen. Sometimes the damage is caused by a heart attack, but it may also be caused by other medical conditions, such as damaged heart valves, high blood pressure or diabetes. Individuals with heart failure suffer from chronic fatigue, memory loss, fluid retention, and breathlessness from even minimal activity, such as walking, shopping or carrying out household chores. They are at increased risk for life-threatening conditions, such as pulmonary edema (where fluid accumulates in the lungs) and sudden cardiac death.

What might it replace?
Lifestyle changes (diet and exercise) combined with various drug treatments are currently used to help manage the symptoms of heart failure. In addition to drug treatments, patients with severe heart failure may have implantable cardioverter defibrillators, pacemakers, or other "heart assist" devices surgically inserted into their chests.

Who might benefit?
Heart disease is the second leading cause of death in Canada (after cancer). Over 33,000 Canadians die as a result of heart disease each year and about 1.4 million Canadians are living with heart disease. Of these, about 500,000 Canadians have heart failure.

How might they benefit?
Stem cells injected into the heart can help it to repair the damage, for example, the massive cell damage that follows a heart attack, and regenerate new tissue that improves the heart's ability to function, increasing patients' wellbeing (e.g., energy level and mental functioning) and allowing them to resume their normal daily activities.

What happens if it isn't funded?
Individuals with heart failure will continue to receive drug therapies and implanted medical devices., and some will remain unable to carry out many activities of normal life.

Technology name: Monoclonal antibody treatment for high cholesterol

What is monoclonal antibody treatment?
Antibodies are proteins made by the immune system that identify and destroy foreign substances in the body (e.g., bacteria). An antibody that targets one specific substance is called a monoclonal antibody (mAb). Alirocumab and Evolocumab are 2 mAbs that have been produced to help treat high cholesterol.

Cholesterol is a fat-like substance that is essential to the functioning of the human body. It occurs in 2 forms: high density lipoprotein cholesterol (HDL-C or "good" cholesterol) and low density lipoprotein cholesterol (LDL-C or "bad" cholesterol). High LDL-C levels can lead to heart disease and increased risk of heart-related events (i.e., heart attack and stroke). A substance in the body called PCSK9 is known to increase levels of the "bad" cholesterol by stopping its breakdown. Alirocumab and Evolucumab target and destroy PCSK9. Both mAb treatments are taken by patients every 2 to 4 weeks for life through a self-administered injection.

What might it replace?
Currently, statins, which prevent the production of cholesterol in the body, are used to treat high cholesterol. Once started, statins usually have to be taken daily for life. Though they are generally well-tolerated, statins have some minor side-effects (e.g., muscle and joint aches, headache, nausea) and some rare, serious side-effects (e.g., muscle problems, type 2 diabetes). Some patients are unable to significantly lower their "bad" cholesterol levels with statins, while others are unable to tolerate effective doses of these drugs. These patients remain at high risk for heart-related events.

Who might benefit?
Alirocumab and Evolocumab may benefit patients who have severe forms of high cholesterol or are at high risk of heart-related events, who are intolerant to statins or whose LDL-C levels are not controlled by statins.

It is estimated that over 7 million Canadians have unhealthy levels of LDL-C ("bad" cholesterol). Approximately 8% are on statins. About half of patients on statins do not achieve their target level of LDL-C, though this may be partially due to dosages being too low.

How might they benefit?
Alirocumab and Evolocumab significantly reduces LDL-C levels in patients, including those who are already taking the highest dose of the most effective statin available. The cholesterol-lowering effects of these mAbs will help to reduce the risk of heart disease, heart attacks and strokes.

In general, Alirocumab and Evolocumab are examples of the innovative work being done to translate genetics research directly into new therapies. Their development has contributed to increased understanding of bodily processes involving cholesterol.

What happens if it isn't funded?
If mAbs are not funded, patients will continue to receive maximal doses of statin therapy, if possible. Those with high LCL-C levels despite the use of statins will remain at high risk for developing heart disease or experiencing heart-related events.

Technology name: Glycerol phenylbutyrate for urea cycle disorder

What is it?
Glycerol phenylbutyrate is a drug that treats people 2 years of age and older with severe urea cycle disorders (UCDs). A urea cycle disorder is a genetic condition where the body does not produce enough of 1 of the 6 enzymes needed to remove ammonia from the blood. When ammonia is allowed to build up in the blood, it becomes toxic, and causes behavior problems, low energy, confusion, and memory problems. Over time, it can cause irreversible brain damage, coma and death. Most people with urea cycle disorders are diagnosed as infants.

Glycerol phenylbutyrate helps prevent a build-up of ammonia in the blood. It does not reverse any brain damage if it has already occurred. Glycerol phenylbutyrate is a liquid, and patients take 3 teaspoons per day for the rest of their lives.

What might it replace?
Currently, patients with severe UCD are treated with a drug called sodium phenylbutyrate. This drug, which comes in pill form,works as well as glycerol phenylbutyrate at preventing ammonia from building up in the blood. However, it tastes and smells very bad. Glycerol phenylbutyrate is an odorless, tasteless liquid. Though many patients crush up the sodium phenylbutyrate pills and take them through a tube in inserted directly into the stomach, those who take them by mouth find them difficult to swallow because they taste so bad. Some patients have to take up to 40 pills a day. Sodium phenylbutyrate also causes an unfavourable body odor in some patients. Their breath and sweat smell musty or like rotten eggs. Patients on either drug still need to follow a strict low-protein diet because digesting protein increases blood ammonia levels.

Who might benefit?
Urea cycle disorders affect 1 in every 35,000 births, which means there are about 1000 patients in Canada with a urea cycle disorder.

How might they benefit?
Glycerol phenylbutyrate is easier to take (3 teaspoons of an odorless colorless liquid compared to 40 pills that taste very bad) and easier for caregivers to help administer. Also, patients with extremely high blood pressure who cannot tolerate the sodium from the sodium phenylbutyrate can be treated with glycerol phenylbutyrate. Patients would not have to deal with the unpleasant body odor associated with the sodium phenylbutyrate and all the ways that it affects sense of self and relationships with others.

What happens if it isn't funded?
If it isn't funded, patients will continue on the sodium formulation of the therapy.

Technology name: Gene therapy for choroideremia

What is it?
Gene therapy is a treatment for choroideremia that involves injecting a healthy copy of a gene into retina cells in the eye to restore normal function of the eye. Choroideremia is a rare genetic condition in which a faulty gene produces faulty proteins that slowly damage the retina of the eye, causing progressive vision loss and eventually complete blindness. Patients with choroideremia usually start to lose their vision in grade school and become legally blind by 40 years of age. Choroideremia affects males and females differently. While females lose some of their vision, males tend to go completely blind.

What might it replace?
There is no other treatment for choroideremia. Standard care is regular monitoring for changes or complications.

Who might benefit?
Choroideremia affects 1 in every 50,000 Canadian men, which means that there are about 700 men with choroideremia in Canada.

How might they benefit?
While gene therapy isn't expected to cure someone who is already blind, it may be able to improve sight in those who are going blind and delay further progression toward blindness. This would allow patients to continue activities that they may have otherwise had to give up as their vision worsened. For example, they may be able to continue to drive, increasing their self-sufficiency and reducing any burden on families and caregivers.

Because this treatment is one of the first gene therapies available to patients, society may benefit from an increase in scientific understanding around how the body responds to the copied gene over time and how to copy other genes for other diseases that have no cure.

What happens if it isn't funded?
If it isn't funded, patients with choroideremia will experience the regular progression toward complete blindness.

Technology name: Dialectical behavior therapy (DBT) for suicide prevention in teenagers

What is it?
Dialectical behavior therapy (DBT) is a type of cognitive behavioural therapy (CBT) that helps people change patterns of behavior that are harmful, like suicidal thinking. It includes 4 components: 1) skills training group to teach behavioural skills through weekly group meetings over 24 weeks, 2) individual treatment, which runs concurrently with skills group and focuses on applying skills to specific challenges, 3) phone coaching that provides in-the-moment coaching on how to use skills to cope with difficult situations that arise in everyday life, and 4) a consultation team to provide support for DBT therapists. The skills training comprises four modules: 1) mindfulness – the practice of being full aware and present in this moment, 2) distress tolerance – how to tolerate pain in difficult situations, 3) interpersonal effectiveness – how to ask for what you want and say no while maintaining self-respect and relationships with others and 4) emotion regulation – how to change emotions that you want to change.

In teenagers with persistent suicidal thoughts, DBT significantly reduces self-reported depression, hopelessness and episodes of deliberate self-harm.

What might it replace?
Current treatments include short-term counselling, medications, problem solving psychotherapy, other forms of CBT, and regular review sessions with a clinical psychologist or psychiatrist. However, they may not be as effective or accessible to everyone, since clinical psychologist services are not covered by public health insurance.

Who might benefit?
In Canada, suicide is the second highest cause of death for youth aged 10-19. Each year, on average, 250 youths die from suicide. Many more attempt suicide. It is estimated that there are 20 suicide attempts for every suicide.

How might they benefit?
Feelings of "wanting to hurt self" or "wanting to kill self" may be significantly reduced, improving quality of life and ability to become a healthy, productive adult. The emotional well-being of parents may also improve, since there is less worry that their child may commit suicide.

What happens if it isn't funded?
Teenagers will continue to have access to services currently available.

Exercise 2 ('Does it matter who benefits and by how much?') Questions

Group 1

Question 1:

Which of the following would you prefer?

	Option 1		Option 2
**Patient Benefit**	Large		Small
**Caregiver Benefit**	Small	Or	Large
**Societal Benefit**	Small		Small

☐ **Option 1** ☐ **Option 2**

Are you certain or uncertain about your choice?

☐ Certain

☐ Uncertain

Question 2:

Which of the following would you prefer?

	Option 1		Option 2
**Patient Benefit**	Small		Medium
**Caregiver Benefit**	Medium	Or	Small
**Societal Benefit**	Small		Small

☐ **Option 1** ☐ **Option 2**

Are you certain or uncertain about your choice?

☐ Certain

☐ Uncertain

Question 3:

Which of the following would you prefer?

	Option 1		Option 2
**Patient Benefit**	Large		Medium
**Caregiver Benefit**	Medium	Or	Large
**Societal Benefit**	Small		Small

☐ **Option 1** ☐ **Option 2**

Are you certain or uncertain about your choice?

☐ Certain

☐ Uncertain

Question 4:

Which of the following would you prefer?

	Option 1		Option 2
**Patient Benefit**	Large		Small
**Caregiver Benefit**	Small	Or	Small
**Societal Benefit**	Small		Large

☐ **Option 1** ☐ **Option 2**

Are you certain or uncertain about your choice?

☐ Certain

☐ Uncertain

Question 5:

Which of the following would you prefer?

	Option 1		Option 2
**Patient Benefit**	Small		Medium
**Caregiver Benefit**	Small	Or	Small
**Societal Benefit**	Medium		Small

☐ **Option 1** ☐ **Option 2**

Are you certain or uncertain about your choice?

☐ Certain

☐ Uncertain

Question 6:

Which of the following would you prefer?

	Option 1		Option 2
**Patient Benefit**	Large		Medium
**Caregiver Benefit**	Small	Or	Small
**Societal Benefit**	Medium		Large

☐ **Option 1** ☐ **Option 2**

Are you certain or uncertain about your choice?

☐ Certain

☐ Uncertain

Question 7:

Which of the following would you prefer?

	Option 1	Or	Option 2
Patient Benefit	Small		Small
Caregiver Benefit	Large	Or	Small
Societal Benefit	Small		Large
	☐ Option 1		☐ Option 2

Are you certain or uncertain about your choice?

☐ Certain
☐ Uncertain

Question 8:

Which of the following would you prefer?

	Option 1	Or	Option 2
Patient Benefit	Small		Small
Caregiver Benefit	Small	Or	Medium
Societal Benefit	Medium		Small
	☐ Option 1		☐ Option 2

Are you certain or uncertain about your choice?

☐ Certain
☐ Uncertain

Question 9:

Which of the following would you prefer?

	Option 1	Or	Option 2
Patient Benefit	Small		Small
Caregiver Benefit	Large	Or	Medium
Societal Benefit	Medium		Large
	☐ Option 1		☐ Option 2

Are you certain or uncertain about your choice?

☐ Certain
☐ Uncertain

Question 10:

Which of the following would you prefer?

	Option 1	Or	Option 2
Patient Benefit	Small		Large
Caregiver Benefit	Medium	Or	Small
Societal Benefit	Large		Medium
	☐ Option 1		☐ Option 2

Are you certain or uncertain about your choice?

☐ Certain
☐ Uncertain

Question 11:

Which of the following would you prefer?

	Option 1	Or	Option 2
Patient Benefit	Small		Large
Caregiver Benefit	Large	Or	Medium
Societal Benefit	Medium		Small
	☐ Option 1		☐ Option 2

Are you certain or uncertain about your choice?

☐ Certain
☐ Uncertain

Question 12:

Which of the following would you prefer?

	Option 1	Or	Option 2
Patient Benefit	Medium		Small
Caregiver Benefit	Small	Or	Large
Societal Benefit	Large		Small
	☐ Option 1		☐ Option 2

Are you certain or uncertain about your choice?

☐ Certain
☐ Uncertain

Question 13:

Which of the following would you prefer?

	Option 1	Or	Option 2
**Patient Benefit**	Medium		Small
**Caregiver Benefit**	Large	Or	Small
**Societal Benefit**	Small		Large

☐ **Option 1** ☐ **Option 2**

Are you certain or uncertain about your choice?

☐ Certain
☐ Uncertain

Question 14:

Which of the following would you prefer?

	Option 1	Or	Option 2
**Patient Benefit**	Small		Medium
**Caregiver Benefit**	Large	Or	Small
**Societal Benefit**	Small		Small

☐ **Option 1** ☐ **Option 2**

Are you certain or uncertain about your choice?

☐ Certain
☐ Uncertain

Question 15:

Which of the following would you prefer?

	Option 1	Or	Option 2
**Patient Benefit**	Medium		Large
**Caregiver Benefit**	Large	Or	Small
**Societal Benefit**	Small		Small

☐ **Option 1** ☐ **Option 2**

Are you certain or uncertain about your choice?

☐ Certain
☐ Uncertain

Question 16:

Which of the following would you prefer?

	Option 1	Or	Option 2
**Patient Benefit**	Small		Medium
**Caregiver Benefit**	Small	Or	Small
**Societal Benefit**	Large		Small

☐ **Option 1** ☐ **Option 2**

Are you certain or uncertain about your choice?

☐ Certain
☐ Uncertain

Question 17:

Which of the following would you prefer?

	Option 1	Or	Option 2
**Patient Benefit**	Medium		Large
**Caregiver Benefit**	Small	Or	Small
**Societal Benefit**	Large		Small

☐ **Option 1** ☐ **Option 2**

Are you certain or uncertain about your choice?

☐ Certain
☐ Uncertain

Question 18:

Which of the following would you prefer?

	Option 1	Or	Option 2
**Patient Benefit**	Small		Medium
**Caregiver Benefit**	Medium	Or	Small
**Societal Benefit**	Large		Small

☐ **Option 1** ☐ **Option 2**

Are you certain or uncertain about your choice?

☐ Certain
☐ Uncertain

Question 19:

Which of the following would you prefer?

	Option 1		Option 2
Patient Benefit	Large		Small
Caregiver Benefit	Small	Or	Medium
Societal Benefit	Small		Small
	☐ Option 1		☐ Option 2

Are you certain or uncertain about your choice?

☐ Certain

☐ Uncertain

Question 20:

Which of the following would you prefer?

	Option 1		Option 2
Patient Benefit	Large		Small
Caregiver Benefit	Medium	Or	Large
Societal Benefit	Small		Small
	☐ Option 1		☐ Option 2

Are you certain or uncertain about your choice?

☐ Certain

☐ Uncertain

Question 21:

Which of the following would you prefer?

	Option 1		Option 2
Patient Benefit	Small		Small
Caregiver Benefit	Small	Or	Medium
Societal Benefit	Large		Small
	☐ Option 1		☐ Option 2

Are you certain or uncertain about your choice?

☐ Certain

☐ Uncertain

Question 22:

Which of the following would you prefer?

	Option 1		Option 2
Patient Benefit	Small		Small
Caregiver Benefit	Medium	Or	Large
Societal Benefit	Large		Small
	☐ Option 1		☐ Option 2

Are you certain or uncertain about your choice?

☐ Certain

☐ Uncertain

Question 23:

Which of the following would you prefer?

	Option 1		Option 2
Patient Benefit	Large		Small
Caregiver Benefit	Small	Or	Medium
Societal Benefit	Large		Small
	☐ Option 1		☐ Option 2

Are you certain or uncertain about your choice?

☐ Certain

☐ Uncertain

Question 24:

Which of the following would you prefer?

	Option 1		Option 2
Patient Benefit	Large		Small
Caregiver Benefit	Small	Or	Small
Societal Benefit	Medium		Large
	☐ Option 1		☐ Option 2

Are you certain or uncertain about your choice?

☐ Certain

☐ Uncertain

Question 25:

Which of the following would you prefer?

	Option 1	Or	Option 2
**Patient Benefit**	Small		Large
**Caregiver Benefit**	Small		Small
**Societal Benefit**	Large		Small

☐ **Option 1** ☐ **Option 2**

Are you certain or uncertain about your choice?

☐ Certain
☐ Uncertain

Question 26:

Which of the following would you prefer?

	Option 1	Or	Option 2
**Patient Benefit**	Small		Small
**Caregiver Benefit**	Large		Small
**Societal Benefit**	Medium		Large

☐ **Option 1** ☐ **Option 2**

Are you certain or uncertain about your choice?

☐ Certain
☐ Uncertain

Question 27:

Which of the following would you prefer?

	Option 1	Or	Option 2
**Patient Benefit**	Small		Small
**Caregiver Benefit**	Large		Small
**Societal Benefit**	Small		Medium

☐ **Option 1** ☐ **Option 2**

Are you certain or uncertain about your choice?

☐ Certain
☐ Uncertain

Question 28:

Which of the following would you prefer?

	Option 1	Or	Option 2
**Patient Benefit**	Large		Small
**Caregiver Benefit**	Medium		Small
**Societal Benefit**	Small		Medium

☐ **Option 1** ☐ **Option 2**

Are you certain or uncertain about your choice?

☐ Certain
☐ Uncertain

Question 29:

Which of the following would you prefer?

	Option 1	Or	Option 2
**Patient Benefit**	Small		Large
**Caregiver Benefit**	Medium		Small
**Societal Benefit**	Large		Medium

☐ **Option 1** ☐ **Option 2**

Are you certain or uncertain about your choice?

☐ Certain
☐ Uncertain

Group 2

Question 1:

Which of the following would you prefer?

	Option 1		Option 2
**Patient Benefit**	Large		Small
**Caregiver Benefit**	Small	Or	Large
**Societal Benefit**	Medium		Medium

☐ **Option 1** ☐ **Option 2**

Are you certain or uncertain about your choice?

☐ Certain
☐ Uncertain

Question 2:

Which of the following would you prefer?

	Option 1		Option 2
**Patient Benefit**	Small		Medium
**Caregiver Benefit**	Medium	Or	Small
**Societal Benefit**	Medium		Medium

☐ **Option 1** ☐ **Option 2**

Are you certain or uncertain about your choice?

☐ Certain
☐ Uncertain

Question 3:

Which of the following would you prefer?

	Option 1		Option 2
**Patient Benefit**	Large		Medium
**Caregiver Benefit**	Medium	Or	Large
**Societal Benefit**	Medium		Medium

☐ **Option 1** ☐ **Option 2**

Are you certain or uncertain about your choice?

☐ Certain
☐ Uncertain

Question 4:

Which of the following would you prefer?

	Option 1		Option 2
**Patient Benefit**	Large		Small
**Caregiver Benefit**	Medium	Or	Medium
**Societal Benefit**	Small		Large

☐ **Option 1** ☐ **Option 2**

Are you certain or uncertain about your choice?

☐ Certain
☐ Uncertain

Question 5:

Which of the following would you prefer?

	Option 1		Option 2
**Patient Benefit**	Small		Medium
**Caregiver Benefit**	Medium	Or	Medium
**Societal Benefit**	Medium		Small

☐ **Option 1** ☐ **Option 2**

Are you certain or uncertain about your choice?

☐ Certain
☐ Uncertain

Question 6:

Which of the following would you prefer?

	Option 1		Option 2
**Patient Benefit**	Large		Medium
**Caregiver Benefit**	Medium	Or	Medium
**Societal Benefit**	Medium		Large

☐ **Option 1** ☐ **Option 2**

Are you certain or uncertain about your choice?

☐ Certain
☐ Uncertain

Question 7:

Which of the following would you prefer?

	Option 1	Or	Option 2
**Patient Benefit**	Medium		Medium
**Caregiver Benefit**	Large	Or	Small
**Societal Benefit**	Small		Large

☐ **Option 1** ☐ **Option 2**

Are you certain or uncertain about your choice?

☐ Certain
☐ Uncertain

Question 8:

Which of the following would you prefer?

	Option 1	Or	Option 2
**Patient Benefit**	Medium		Medium
**Caregiver Benefit**	Small	Or	Medium
**Societal Benefit**	Medium		Small

☐ **Option 1** ☐ **Option 2**

Are you certain or uncertain about your choice?

☐ Certain
☐ Uncertain

Question 9:

Which of the following would you prefer?

	Option 1	Or	Option 2
**Patient Benefit**	Medium		Medium
**Caregiver Benefit**	Large	Or	Medium
**Societal Benefit**	Medium		Large

☐ **Option 1** ☐ **Option 2**

Are you certain or uncertain about your choice?

☐ Certain
☐ Uncertain

Question 10:

Which of the following would you prefer?

	Option 1	Or	Option 2
**Patient Benefit**	Small		Large
**Caregiver Benefit**	Medium	Or	Small
**Societal Benefit**	Large		Medium

☐ **Option 1** ☐ **Option 2**

Are you certain or uncertain about your choice?

☐ Certain
☐ Uncertain

Question 11:

Which of the following would you prefer?

	Option 1	Or	Option 2
**Patient Benefit**	Large		Small
**Caregiver Benefit**	Small	Or	Large
**Societal Benefit**	Medium		Small

☐ **Option 1** ☐ **Option 2**

Are you certain or uncertain about your choice?

☐ Certain
☐ Uncertain

Question 12:

Which of the following would you prefer?

	Option 1	Or	Option 2
**Patient Benefit**	Medium		Small
**Caregiver Benefit**	Small	Or	Large
**Societal Benefit**	Large		Small

☐ **Option 1** ☐ **Option 2**

Are you certain or uncertain about your choice?

☐ Certain
☐ Uncertain

Question 13:

Which of the following would you prefer?

	Option 1	Or	Option 2
**Patient Benefit**	Medium		Small
**Caregiver Benefit**	Large	Or	Small
**Societal Benefit**	Small		Large
	☐ **Option 1**		☐ **Option 2**

Are you certain or uncertain about your choice?

☐ Certain
☐ Uncertain

Question 14:

Which of the following would you prefer?

	Option 1	Or	Option 2
**Patient Benefit**	Small		Medium
**Caregiver Benefit**	Large	Or	Small
**Societal Benefit**	Medium		Medium
	☐ **Option 1**		☐ **Option 2**

Are you certain or uncertain about your choice?

☐ Certain
☐ Uncertain

Question 15:

Which of the following would you prefer?

	Option 1	Or	Option 2
**Patient Benefit**	Medium		Large
**Caregiver Benefit**	Large	Or	Small
**Societal Benefit**	Medium		Medium
	☐ **Option 1**		☐ **Option 2**

Are you certain or uncertain about your choice?

☐ Certain
☐ Uncertain

Question 16:

Which of the following would you prefer?

	Option 1	Or	Option 2
**Patient Benefit**	Small		Medium
**Caregiver Benefit**	Medium	Or	Medium
**Societal Benefit**	Large		Small
	☐ **Option 1**		☐ **Option 2**

Are you certain or uncertain about your choice?

☐ Certain
☐ Uncertain

Question 17:

Which of the following would you prefer?

	Option 1	Or	Option 2
**Patient Benefit**	Medium		Large
**Caregiver Benefit**	Medium	Or	Medium
**Societal Benefit**	Large		Small
	☐ **Option 1**		☐ **Option 2**

Are you certain or uncertain about your choice?

☐ Certain
☐ Uncertain

Question 18:

Which of the following would you prefer?

	Option 1	Or	Option 2
**Patient Benefit**	Small		Medium
**Caregiver Benefit**	Large	Or	Small
**Societal Benefit**	Large		Small
	☐ **Option 1**		☐ **Option 2**

Are you certain or uncertain about your choice?

☐ Certain
☐ Uncertain

Question 19:

Which of the following would you prefer?

	Option 1		Option 2
**Patient Benefit**	Large		Small
**Caregiver Benefit**	Small	Or	Medium
**Societal Benefit**	Medium		Medium
	☐ **Option 1**		☐ **Option 2**

Are you certain or uncertain about your choice?

☐ Certain
☐ Uncertain

Question 20:

Which of the following would you prefer?

	Option 1		Option 2
**Patient Benefit**	Large		Small
**Caregiver Benefit**	Medium	Or	Large
**Societal Benefit**	Medium		Medium
	☐ **Option 1**		☐ **Option 2**

Are you certain or uncertain about your choice?

☐ Certain
☐ Uncertain

Question 21:

Which of the following would you prefer?

	Option 1		Option 2
**Patient Benefit**	Medium		Medium
**Caregiver Benefit**	Small	Or	Medium
**Societal Benefit**	Large		Small
	☐ **Option 1**		☐ **Option 2**

Are you certain or uncertain about your choice?

☐ Certain
☐ Uncertain

Question 22:

Which of the following would you prefer?

	Option 1		Option 2
**Patient Benefit**	Medium		Medium
**Caregiver Benefit**	Medium	Or	Large
**Societal Benefit**	Large		Small
	☐ **Option 1**		☐ **Option 2**

Are you certain or uncertain about your choice?

☐ Certain
☐ Uncertain

Question 23:

Which of the following would you prefer?

	Option 1		Option 2
**Patient Benefit**	Medium		Small
**Caregiver Benefit**	Small	Or	Medium
**Societal Benefit**	Large		Small
	☐ **Option 1**		☐ **Option 2**

Are you certain or uncertain about your choice?

☐ Certain
☐ Uncertain

Question 24:

Which of the following would you prefer?

	Option 1		Option 2
**Patient Benefit**	Large		Small
**Caregiver Benefit**	Medium	Or	Medium
**Societal Benefit**	Medium		Large
	☐ **Option 1**		☐ **Option 2**

Are you certain or uncertain about your choice?

☐ Certain
☐ Uncertain

Question 25:

Which of the following would you prefer?

	Option 1		Option 2
**Patient Benefit**	Small		Large
**Caregiver Benefit**	Medium	Or	Medium
**Societal Benefit**	Large		Small

☐ **Option 1** ☐ **Option 2**

Are you certain or uncertain about your choice?

☐ Certain
☐ Uncertain

Question 26:

Which of the following would you prefer?

	Option 1		Option 2
**Patient Benefit**	Medium		Medium
**Caregiver Benefit**	Large	Or	Small
**Societal Benefit**	Medium		Large

☐ **Option 1** ☐ **Option 2**

Are you certain or uncertain about your choice?

☐ Certain
☐ Uncertain

Question 27:

Which of the following would you prefer?

	Option 1		Option 2
**Patient Benefit**	Medium		Medium
**Caregiver Benefit**	Large	Or	Small
**Societal Benefit**	Small		Medium

☐ **Option 1** ☐ **Option 2**

Are you certain or uncertain about your choice?

☐ Certain
☐ Uncertain

Question 28:

Which of the following would you prefer?

	Option 1		Option 2
**Patient Benefit**	Large		Small
**Caregiver Benefit**	Medium	Or	Small
**Societal Benefit**	Small		Medium

☐ **Option 1** ☐ **Option 2**

Are you certain or uncertain about your choice?

☐ Certain
☐ Uncertain

Question 29:

Which of the following would you prefer?

	Option 1		Option 2
**Patient Benefit**	Small		Large
**Caregiver Benefit**	Medium	Or	Small
**Societal Benefit**	Large		Medium

☐ **Option 1** ☐ **Option 2**

Are you certain or uncertain about your choice?

☐ Certain
☐ Uncertain

Group 3

Question 1:

Which of the following would you prefer?

**Patient Benefit**	Large		Small
**Caregiver Benefit**	Small	Or	Large
**Societal Benefit**	Large		Large
	☐ **Option 1**		☐ **Option 2**

Are you certain or uncertain about your choice?

☐ Certain

☐ Uncertain

Question 2:

Which of the following would you prefer?

**Patient Benefit**	Small		Medium
**Caregiver Benefit**	Medium	Or	Small
**Societal Benefit**	Large		Large
	☐ **Option 1**		☐ **Option 2**

Are you certain or uncertain about your choice?

☐ Certain

☐ Uncertain

Question 3:

Which of the following would you prefer?

**Patient Benefit**	Large		Medium
**Caregiver Benefit**	Medium	Or	Large
**Societal Benefit**	Large		Large
	☐ **Option 1**		☐ **Option 2**

Are you certain or uncertain about your choice?

☐ Certain

☐ Uncertain

Question 4:

Which of the following would you prefer?

**Patient Benefit**	Large		Small
**Caregiver Benefit**	Large	Or	Large
**Societal Benefit**	Small		Large
	☐ **Option 1**		☐ **Option 2**

Are you certain or uncertain about your choice?

☐ Certain

☐ Uncertain

Question 5:

Which of the following would you prefer?

**Patient Benefit**	Small		Medium
**Caregiver Benefit**	Large	Or	Large
**Societal Benefit**	Medium		Small
	☐ **Option 1**		☐ **Option 2**

Are you certain or uncertain about your choice?

☐ Certain

☐ Uncertain

Question 6:

Which of the following would you prefer?

**Patient Benefit**	Large		Medium
**Caregiver Benefit**	Large	Or	Large
**Societal Benefit**	Medium		Large
	☐ **Option 1**		☐ **Option 2**

Are you certain or uncertain about your choice?

☐ Certain

☐ Uncertain

Question 7:

Which of the following would you prefer?

	Option 1	Or	Option 2
**Patient Benefit**	Large		Large
**Caregiver Benefit**	Large	Or	Small
**Societal Benefit**	Small		Large

☐ **Option 1** ☐ **Option 2**

Are you certain or uncertain about your choice?

☐ Certain
☐ Uncertain

Question 8:

Which of the following would you prefer?

	Option 1	Or	Option 2
**Patient Benefit**	Large		Large
**Caregiver Benefit**	Small	Or	Medium
**Societal Benefit**	Medium		Small

☐ **Option 1** ☐ **Option 2**

Are you certain or uncertain about your choice?

☐ Certain
☐ Uncertain

Question 9:

Which of the following would you prefer?

	Option 1	Or	Option 2
**Patient Benefit**	Large		Large
**Caregiver Benefit**	Large	Or	Medium
**Societal Benefit**	Medium		Large

☐ **Option 1** ☐ **Option 2**

Are you certain or uncertain about your choice?

☐ Certain
☐ Uncertain

Question 10:

Which of the following would you prefer?

	Option 1	Or	Option 2
**Patient Benefit**	Small		Large
**Caregiver Benefit**	Medium	Or	Small
**Societal Benefit**	Large		Medium

☐ **Option 1** ☐ **Option 2**

Are you certain or uncertain about your choice?

☐ Certain
☐ Uncertain

Question 11:

Which of the following would you prefer?

	Option 1	Or	Option 2
**Patient Benefit**	Large		Small
**Caregiver Benefit**	Medium	Or	Small
**Societal Benefit**	Small		Large

☐ **Option 1** ☐ **Option 2**

Are you certain or uncertain about your choice?

☐ Certain
☐ Uncertain

Question 12:

Which of the following would you prefer?

	Option 1	Or	Option 2
**Patient Benefit**	Medium		Small
**Caregiver Benefit**	Small	Or	Large
**Societal Benefit**	Large		Small

☐ **Option 1** ☐ **Option 2**

Are you certain or uncertain about your choice?

☐ Certain
☐ Uncertain

Question 13:

Which of the following would you prefer?

	Option 1		Option 2
Patient Benefit	Medium	Or	Small
Caregiver Benefit	Large		Small
Societal Benefit	Small		Large

☐ Option 1 ☐ Option 2

Are you certain or uncertain about your choice?

☐ Certain
☐ Uncertain

Question 14:

Which of the following would you prefer?

	Option 1		Option 2
Patient Benefit	Small	Or	Medium
Caregiver Benefit	Large		Small
Societal Benefit	Large		Large

☐ Option 1 ☐ Option 2

Are you certain or uncertain about your choice?

☐ Certain
☐ Uncertain

Question 15:

Which of the following would you prefer?

	Option 1		Option 2
Patient Benefit	Medium	Or	Large
Caregiver Benefit	Large		Small
Societal Benefit	Large		Large

☐ Option 1 ☐ Option 2

Are you certain or uncertain about your choice?

☐ Certain
☐ Uncertain

Question 16:

Which of the following would you prefer?

	Option 1		Option 2
Patient Benefit	Small	Or	Medium
Caregiver Benefit	Large		Large
Societal Benefit	Large		Small

☐ Option 1 ☐ Option 2

Are you certain or uncertain about your choice?

☐ Certain
☐ Uncertain

Question 17:

Which of the following would you prefer?

	Option 1		Option 2
Patient Benefit	Medium	Or	Large
Caregiver Benefit	Large		Large
Societal Benefit	Large		Small

☐ Option 1 ☐ Option 2

Are you certain or uncertain about your choice?

☐ Certain
☐ Uncertain

Question 18:

Which of the following would you prefer?

	Option 1		Option 2
Patient Benefit	Small	Or	Medium
Caregiver Benefit	Medium		Small
Societal Benefit	Large		Small

☐ Option 1 ☐ Option 2

Are you certain or uncertain about your choice?

☐ Certain
☐ Uncertain

Question 19:

Which of the following would you prefer?

	Option 1	Or	Option 2
**Patient Benefit**	Large		Small
**Caregiver Benefit**	Small	Or	Medium
**Societal Benefit**	Large		Large

☐ **Option 1** ☐ **Option 2**

Are you certain or uncertain about your choice?

☐ Certain
☐ Uncertain

Question 20:

Which of the following would you prefer?

	Option 1	Or	Option 2
**Patient Benefit**	Large		Small
**Caregiver Benefit**	Medium	Or	Large
**Societal Benefit**	Large		Large

☐ **Option 1** ☐ **Option 2**

Are you certain or uncertain about your choice?

☐ Certain
☐ Uncertain

Question 21:

Which of the following would you prefer?

	Option 1	Or	Option 2
**Patient Benefit**	Large		Large
**Caregiver Benefit**	Small	Or	Medium
**Societal Benefit**	Large		Small

☐ **Option 1** ☐ **Option 2**

Are you certain or uncertain about your choice?

☐ Certain
☐ Uncertain

Question 22:

Which of the following would you prefer?

	Option 1	Or	Option 2
**Patient Benefit**	Large		Large
**Caregiver Benefit**	Medium	Or	Large
**Societal Benefit**	Large		Small

☐ **Option 1** ☐ **Option 2**

Are you certain or uncertain about your choice?

☐ Certain
☐ Uncertain

Question 23:

Which of the following would you prefer?

	Option 1	Or	Option 2
**Patient Benefit**	Medium		Small
**Caregiver Benefit**	Small	Or	Medium
**Societal Benefit**	Large		Small

☐ **Option 1** ☐ **Option 2**

Are you certain or uncertain about your choice?

☐ Certain
☐ Uncertain

Question 24:

Which of the following would you prefer?

	Option 1	Or	Option 2
**Patient Benefit**	Large		Small
**Caregiver Benefit**	Large	Or	Large
**Societal Benefit**	Medium		Large

☐ **Option 1** ☐ **Option 2**

Are you certain or uncertain about your choice?

☐ Certain
☐ Uncertain

Question 25:

Which of the following would you prefer?

	Option 1		Option 2
**Patient Benefit**	Small		Large
**Caregiver Benefit**	Large	Or	Large
**Societal Benefit**	Large		Small
	☐ **Option 1**		☐ **Option 2**

Are you certain or uncertain about your choice?

☐ Certain
☐ Uncertain

Question 26:

Which of the following would you prefer?

	Option 1		Option 2
**Patient Benefit**	Large		Large
**Caregiver Benefit**	Large	Or	Small
**Societal Benefit**	Medium		Large
	☐ **Option 1**		☐ **Option 2**

Are you certain or uncertain about your choice?

☐ Certain
☐ Uncertain

Question 27:

Which of the following would you prefer?

	Option 1		Option 2
**Patient Benefit**	Large		Large
**Caregiver Benefit**	Large	Or	Small
**Societal Benefit**	Small		Medium
	☐ **Option 1**		☐ **Option 2**

Are you certain or uncertain about your choice?

☐ Certain
☐ Uncertain

Question 28:

Which of the following would you prefer?

	Option 1		Option 2
**Patient Benefit**	Large		Small
**Caregiver Benefit**	Large	Or	Small
**Societal Benefit**	Small		Medium
	☐ **Option 1**		☐ **Option 2**

Are you certain or uncertain about your choice?

☐ Certain
☐ Uncertain

Question 29:

Which of the following would you prefer?

	Option 1		Option 2
**Patient Benefit**	Small		Large
**Caregiver Benefit**	Medium	Or	Small
**Societal Benefit**	Large		Medium
	☐ **Option 1**		☐ **Option 2**

Are you certain or uncertain about your choice?

☐ Certain
☐ Uncertain

Exercise 3 ('How should differences in health gains affect our choices?') Questions

Group 1

Question 1:

Which of the following would you prefer?

	Option 1 Current	Option 1 Gain		Option 2 Current	Option 2 Gain
Life Expectancy	Less than 1 year	1 to 5 years		Less than 1 year	No change
Physical Functioning	Severely Impaired	No change		Moderately Impaired	No change
Pain & Discomfort	Moderate	Mild	Or	Mild	No change
Cognitive Functioning	Mildly Impaired	No change		Moderately Impaired	Mildly Impaired
Depression & Anxiety	Severe	Moderate		Severe	Mild

☐ Option 1 ☐ Option 2

Would you change your choice if the number of patients affected were different?

☐ Yes
☐ No

Question 2:

Which of the following would you prefer?

	Option 1 Current	Option 1 Gain		Option 2 Current	Option 2 Gain
Life Expectancy	Less than 1 year	5 years or more		1 to 5 years	5 years or more
Physical Functioning	Severely Impaired	Moderately Impaired		Mildly Impaired	No change
Pain & Discomfort	Moderate	Mild	Or	Extreme	Mild
Cognitive Functioning	Moderately Impaired	No change		Severely Impaired	Moderately Impaired
Depression & Anxiety	Moderate	No change		Moderate	Mild

☐ Option 1 ☐ Option 2

Would you change your choice if the number of patients affected were different?

☐ Yes
☐ No

Question 3:

Which of the following would you prefer?

	Option 1 Current	Option 1 Gain		Option 2 Current	Option 2 Gain
Life Expectancy	1 to 5 years	5 years or more		5 years or more	No change
Physical Functioning	Mildly Impaired	No change		Severely Impaired	Mildly Impaired
Pain & Discomfort	Extreme	No change	Or	Extreme	Moderate
Cognitive Functioning	Severely Impaired	Mildly Impaired		Severely Impaired	No change
Depression & Anxiety	Moderate	No change		Severe	No change

☐ Option 1 ☐ Option 2

Would you change your choice if the number of patients affected was different?

☐ Yes
☐ No

Question 4:

Which of the following would you prefer?

	Option 1 Current	Option 1 Gain		Option 2 Current	Option 2 Gain
Life Expectancy	5 years or more	No change		Less than 1 year	1 to 5 years
Physical Functioning	Moderately Impaired	Mildly Impaired		Severely Impaired	Moderately Impaired
Pain & Discomfort	Moderate	No change	Or	Extreme	No change
Cognitive Functioning	Moderately Impaired	No change		Severely Impaired	Moderately Impaired
Depression & Anxiety	Moderate	Mild		Severe	Mild

☐ Option 1 ☐ Option 2

Would you change your choice if the number of patients affected was different?

☐ Yes
☐ No

Question 5:

Which of the following would you prefer?

	Option 1 Current	Option 1 Gain		Option 2 Current	Option 2 Gain
Life Expectancy	Less than 1 year	5 years or more		1 to 5 years	No change
Physical Functioning	Mildly Impaired	No change		Moderately Impaired	Mildly Impaired
Pain & Discomfort	Extreme	No change	Or	Moderate	No change
Cognitive Functioning	Mildly Impaired	No change		Severely Impaired	Moderately Impaired
Depression & Anxiety	Moderate	No change		Severe	Moderate

☐ Option 1 ☐ Option 2

Would you change your choice if the number of patients affected was different?

☐ Yes
☐ No

Question 6:

Which of the following would you prefer?

	Option 1 Current	Option 1 Gain		Option 2 Current	Option 2 Gain
Life Expectancy	Less than 1 year	1 to 5 years		1 to 5 years	5 years or more
Physical Functioning	Moderately Impaired	Mildly Impaired		Severely Impaired	Moderately Impaired
Pain & Discomfort	Extreme	Mild	Or	Mild	No change
Cognitive Functioning	Moderately Impaired	No change		Severely Impaired	Mildly Impaired
Depression & Anxiety	Severe	No change		Moderate	Mild

☐ Option 1 ☐ Option 2

Would you change your choice if the number of patients affected was different?

☐ Yes
☐ No

Question 7:

Which of the following would you prefer?

	Option 1: Current	Option 1: Gain		Option 2: Current	Option 2: Gain
Life Expectancy	1 to 5 years	5 years or more		Less than 1 year	1 to 5 years
Physical Functioning	Mildly Impaired	No change		Severely Impaired	Moderately Impaired
Pain & Discomfort	Extreme	No change	Or	Moderate	Mild
Cognitive Functioning	Severely Impaired	Mildly Impaired		Moderately Impaired	Mildly Impaired
Depression & Anxiety	Severe	Mild		Severe	No change

☐ Option 1 ☐ Option 2

Would you change your choice if the number of patients affected was different?

☐ Yes
☐ No

Question 8:

Which of the following would you prefer?

	Option 1: Current	Option 1: Gain		Option 2: Current	Option 2: Gain
Life Expectancy	5 years or more	No change		Less than 1 year	5 years or more
Physical Functioning	Moderately Impaired	Mildly Impaired		Severely Impaired	Mildly Impaired
Pain & Discomfort	Extreme	Moderate	Or	Moderate	No change
Cognitive Functioning	Mildly Impaired	No change		Severely Impaired	Moderately Impaired
Depression & Anxiety	Moderate	Mild		Moderate	No change

☐ Option 1 ☐ Option 2

Would you change your choice if the number of patients affected was different?

☐ Yes
☐ No

Question 9:

Which of the following would you prefer?

	Option 1: Current	Option 1: Gain		Option 2: Current	Option 2: Gain
Life Expectancy	Less than 1 year	1 to 5 years		1 to 5 years	5 years or more
Physical Functioning	Moderately Impaired	Mildly Impaired		Severely Impaired	No change
Pain & Discomfort	Moderate	No change	Or	Extreme	Mild
Cognitive Functioning	Moderately Impaired	Mildly Impaired		Moderately Impaired	No change
Depression & Anxiety	Mild	No change		Moderate	Mild

☐ Option 1 ☐ Option 2

Would you change your choice if the number of patients affected was different?

☐ Yes
☐ No

Question 10:

Which of the following would you prefer?

	Option 1: Current	Option 1: Gain		Option 2: Current	Option 2: Gain
Life Expectancy	Less than 1 year	No change		Less than 1 year	5 years or more
Physical Functioning	Moderately Impaired	No change		Mildly Impaired	No change
Pain & Discomfort	Extreme	No change	Or	Extreme	Mild
Cognitive Functioning	Moderately Impaired	No change		Mildly Impaired	No change
Depression & Anxiety	Moderate	Mild		Severe	Mild

☐ Option 1 ☐ Option 2

Would you change your choice if the number of patients affected was different?

☐ Yes
☐ No

Question 11:

Which of the following would you prefer?

	Option 1: Current	Option 1: Gain		Option 2: Current	Option 2: Gain
Life Expectancy	Less than 1 year	No change		5 years or more	No change
Physical Functioning	Severely Impaired	No change		Moderately Impaired	No change
Pain & Discomfort	Extreme	No change	Or	Moderate	Mild
Cognitive Functioning	Severely Impaired	Mildly Impaired		Mildly Impaired	No change
Depression & Anxiety	Mild	No change		Severe	Mild

☐ Option 1 ☐ Option 2

Would you change your choice if the number of patients affected was different?

☐ Yes
☐ No

Question 12:

Which of the following would you prefer?

	Option 1: Current	Option 1: Gain		Option 2: Current	Option 2: Gain
Life Expectancy	Less than 1 year	No change		Less than 1 year	1 to 5 years
Physical Functioning	Severely Impaired	Moderately Impaired		Severely Impaired	Mildly Impaired
Pain & Discomfort	Extreme	Mild	Or	Moderate	No change
Cognitive Functioning	Moderately Impaired	No change		Severely Impaired	Mildly Impaired
Depression & Anxiety	Moderate	Mild		Severe	Moderate

☐ Option 1 ☐ Option 2

Would you change your choice if the number of patients affected was different?

☐ Yes
☐ No

Question 13:

Which of the following would you prefer?

	Option 1 Current	Option 1 Gain		Option 2 Current	Option 2 Gain
Life Expectancy	1 to 5 years	5 years or more		Less than 1 year	No change
Physical Functioning	Severely Impaired	Moderately Impaired		Severely Impaired	Mildly Impaired
Pain & Discomfort	Extreme	Mild	Or	Extreme	Moderate
Cognitive Functioning	Moderately Impaired	Mildly Impaired		Moderately Impaired	No change
Depression & Anxiety	Severe	Moderate		Severe	Mild

☐ Option 1 ☐ Option 2

Would you change your choice if the number of patients affected was different?

☐ Yes
☐ No

Question 14:

Which of the following would you prefer?

	Option 1 Current	Option 1 Gain		Option 2 Current	Option 2 Gain
Life Expectancy	1 to 5 years	No change		Less than 1 year	1 to 5 years
Physical Functioning	Moderately Impaired	Mildly Impaired		Moderately Impaired	No change
Pain & Discomfort	Extreme	No change	Or	Extreme	Mild
Cognitive Functioning	Moderately Impaired	Mildly Impaired		Severely Impaired	Moderately Impaired
Depression & Anxiety	Severe	Moderate		Moderate	Mild

☐ Option 1 ☐ Option 2

Would you change your choice if the number of patients affected was different?

☐ Yes
☐ No

Question 15:

Which of the following would you prefer?

	Option 1 Current	Option 1 Gain		Option 2 Current	Option 2 Gain
Life Expectancy	5 years or more	No change		Less than 1 year	No change
Physical Functioning	Moderately Impaired	Mildly Impaired		Severely Impaired	Mildly Impaired
Pain & Discomfort	Mild	No change	Or	Extreme	No change
Cognitive Functioning	Severely Impaired	Mildly Impaired		Severely Impaired	No change
Depression & Anxiety	Severe	No change		Moderate	No change

☐ Option 1 ☐ Option 2

Would you change your choice if the number of patients affected was different?

☐ Yes
☐ No

Question 16:

Which of the following would you prefer?

	Option 1 Current	Option 1 Gain		Option 2 Current	Option 2 Gain
Life Expectancy	1 to 5 years	No change		1 to 5 years	5 years or more
Physical Functioning	Mildly Impaired	No change		Severely Impaired	Mildly Impaired
Pain & Discomfort	Mild	No change	Or	Extreme	Mild
Cognitive Functioning	Severely Impaired	No change		Moderately Impaired	Mildly Impaired
Depression & Anxiety	Severe	Mild		Severe	No change

☐ Option 1 ☐ Option 2

Would you change your choice if the number of patients affected was different?

☐ Yes
☐ No

Question 17:

Which of the following would you prefer?

	Option 1 Current	Option 1 Gain		Option 2 Current	Option 2 Gain
Life Expectancy	1 to 5 years	No change		Less than 1 year	5 years or more
Physical Functioning	Severely Impaired	Moderately Impaired		Severely Impaired	Mildly Impaired
Pain & Discomfort	Extreme	No change	Or	Moderate	Mild
Cognitive Functioning	Severely Impaired	Moderately Impaired		Severely Impaired	Mildly Impaired
Depression & Anxiety	Severe	No change		Severe	Mild

☐ Option 1 ☐ Option 2

Would you change your choice if the number of patients affected was different?

☐ Yes
☐ No

Question 18:

Which of the following would you prefer?

	Option 1 Current	Option 1 Gain		Option 2 Current	Option 2 Gain
Life Expectancy	Less than 1 year	No change		1 to 5 years	No change
Physical Functioning	Mildly Impaired	No change		Moderately Impaired	No change
Pain & Discomfort	Extreme	Moderate	Or	Extreme	Mild
Cognitive Functioning	Severely Impaired	Moderately Impaired		Severely Impaired	Mildly Impaired
Depression & Anxiety	Severe	Moderate		Moderate	Mild

☐ Option 1 ☐ Option 2

Would you change your choice if the number of patients affected was different?

☐ Yes
☐ No

Question 19:

Which of the following would you prefer?

	Current	Gain		Current	Gain
Life Expectancy	1 to 5 years	No change		5 years or more	No change
Physical Functioning	Severely Impaired	No change		Moderately Impaired	No change
Pain & Discomfort	Mild	No change	Or	Extreme	No change
Cognitive Functioning	Severely Impaired	No change		Severely Impaired	Moderately Impaired
Depression & Anxiety	Mild	No change		Severe	No change
	☐ Option 1			☐ Option 2	

Would you change your choice if the number of patients affected was different?

☐ Yes
☐ No

Question 20:

Which of the following would you prefer?

	Current	Gain		Current	Gain
Life Expectancy	1 to 5 years	5 years or more		5 years or more	No change
Physical Functioning	Severely Impaired	Mildly Impaired		Mildly Impaired	No change
Pain & Discomfort	Extreme	Mild	Or	Extreme	No change
Cognitive Functioning	Mildly Impaired	No change		Severely Impaired	Moderately Impaired
Depression & Anxiety	Severe	Mild		Moderate	Mild
	☐ Option 1			☐ Option 2	

Would you change your choice if the number of patients affected was different?

☐ Yes
☐ No

Question 21:

Which of the following would you prefer?

	Current	Gain		Current	Gain
Life Expectancy	5 years or more	No change		1 to 5 years	No change
Physical Functioning	Severely Impaired	Mildly Impaired		Severely Impaired	No change
Pain & Discomfort	Moderate	No change	Or	Moderate	Mild
Cognitive Functioning	Moderately Impaired	No change		Mildly Impaired	No change
Depression & Anxiety	Severe	Moderate		Severe	No change
	☐ Option 1			☐ Option 2	

Would you change your choice if the number of patients affected was different?

☐ Yes
☐ No

Question 22:

Which of the following would you prefer?

	Current	Gain		Current	Gain
Life Expectancy	Less than 1 year	5 years or more		Less than 1 year	No change
Physical Functioning	Severely Impaired	No change		Severely Impaired	Moderately Impaired
Pain & Discomfort	Extreme	Moderate	Or	Mild	No change
Cognitive Functioning	Moderately Impaired	Mildly Impaired		Severely Impaired	Mildly Impaired
Depression & Anxiety	Mild	No change		Severe	Moderate
	☐ Option 1			☐ Option 2	

Would you change your choice if the number of patients affected was different?

☐ Yes
☐ No

Question 23:

Which of the following would you prefer?

	Current	Gain		Current	Gain
Life Expectancy	1 to 5 years	No change		Less than 1 year	5 years or more
Physical Functioning	Moderately Impaired	No change		Mildly Impaired	No change
Pain & Discomfort	Extreme	Moderate	Or	Moderate	No change
Cognitive Functioning	Severely Impaired	Mildly Impaired		Severely Impaired	Moderately Impaired
Depression & Anxiety	Severe	Moderate		Mild	No change
	☐ Option 1			☐ Option 2	

Would you change your choice if the number of patients affected was different?

☐ Yes
☐ No

Question 24:

Which of the following would you prefer?

	Current	Gain		Current	Gain
Life Expectancy	Less than 1 year	1 to 5 years		5 years or more	No change
Physical Functioning	Severely Impaired	Moderately Impaired		Moderately Impaired	Mildly Impaired
Pain & Discomfort	Extreme	No change	Or	Moderate	No change
Cognitive Functioning	Moderately Impaired	No change		Severely Impaired	Mildly Impaired
Depression & Anxiety	Severe	Mild		Moderate	No change
	☐ Option 1			☐ Option 2	

Would you change your choice if the number of patients affected was different?

☐ Yes
☐ No

Question 25:

Which of the following would you prefer?

	Option 1: Current	Option 1: Gain		Option 2: Current	Option 2: Gain
Life Expectancy	Less than 1 year	1 to 5 years		Less than 1 year	5 years or more
Physical Functioning	Severely Impaired	Mildly Impaired		Severely Impaired	Moderately Impaired
Pain & Discomfort	Mild	No change	Or	Moderate	Mild
Cognitive Functioning	Mildly Impaired	No change		Moderately Impaired	Mildly Impaired
Depression & Anxiety	Moderate	Mild		Severe	Moderate

☐ Option 1 ☐ Option 2

Would you change your choice if the number of patients affected was different?

☐ Yes
☐ No

Question 26:

Which of the following would you prefer?

	Option 1: Current	Option 1: Gain		Option 2: Current	Option 2: Gain
Life Expectancy	1 to 5 years	5 years or more		Less than 1 year	5 years or more
Physical Functioning	Severely Impaired	No change		Mildly Impaired	No change
Pain & Discomfort	Moderate	Mild	Or	Extreme	No change
Cognitive Functioning	Moderately Impaired	No change		Severely Impaired	Mildly Impaired
Depression & Anxiety	Moderate	No change		Mild	No change

☐ Option 1 ☐ Option 2

Would you change your choice if the number of patients affected was different?

☐ Yes
☐ No

Question 27:

Which of the following would you prefer?

	Option 1: Current	Option 1: Gain		Option 2: Current	Option 2: Gain
Life Expectancy	Less than 1 year	5 years or more		5 years or more	No change
Physical Functioning	Mildly Impaired	No change		Severely Impaired	No change
Pain & Discomfort	Mild	No change	Or	Moderate	No change
Cognitive Functioning	Mildly Impaired	No change		Severely Impaired	Mildly Impaired
Depression & Anxiety	Severe	Moderate		Severe	No change

☐ Option 1 ☐ Option 2

Would you change your choice if the number of patients affected was different?

☐ Yes
☐ No

Question 28:

Which of the following would you prefer?

	Option 1: Current	Option 1: Gain		Option 2: Current	Option 2: Gain
Life Expectancy	Less than 1 year	1 to 5 years		5 years or more	No change
Physical Functioning	Severely Impaired	No change		Severely Impaired	Moderately Impaired
Pain & Discomfort	Mild	No change	Or	Moderate	No change
Cognitive Functioning	Moderately Impaired	No change		Mildly Impaired	No change
Depression & Anxiety	Severe	Mild		Severe	Moderate

☐ Option 1 ☐ Option 2

Would you change your choice if the number of patients affected was different?

☐ Yes
☐ No

Question 29:

Which of the following would you prefer?

	Option 1: Current	Option 1: Gain		Option 2: Current	Option 2: Gain
Life Expectancy	Less than 1 year	5 years or more		Less than 1 year	1 to 5 years
Physical Functioning	Moderately Impaired	No change		Severely Impaired	Mildly Impaired
Pain & Discomfort	Moderate	No change	Or	Extreme	No change
Cognitive Functioning	Severely Impaired	Moderately Impaired		Severely Impaired	Mildly Impaired
Depression & Anxiety	Moderate	Mild		Moderate	No change

☐ Option 1 ☐ Option 2

Would you change your choice if the number of patients affected was different?

☐ Yes
☐ No

Question 30:

Which of the following would you prefer?

	Option 1: Current	Option 1: Gain		Option 2: Current	Option 2: Gain
Life Expectancy	Less than 1 year	No change		Less than 1 year	5 years or more
Physical Functioning	Moderately Impaired	No change		Mildly Impaired	No change
Pain & Discomfort	Extreme	No change	Or	Extreme	Mild
Cognitive Functioning	Moderately Impaired	No change		Mildly Impaired	No change
Depression & Anxiety	Moderate	Mild		Severe	Mild

☐ Option 1 ☐ Option 2

Would you change your choice if the number of patients affected was different?

☐ Yes
☐ No

Group 2

Question 1:

Which of the following would you prefer?

	Option 1 Current	Option 1 Gain		Option 2 Current	Option 2 Gain
Life Expectancy	Less than 1 year	1 to 5 years		Less than 1 year	No change
Physical Functioning	Severely Impaired	No change		Moderately Impaired	No change
Pain & Discomfort	Moderate	Mild	Or	Mild	No change
Cognitive Functioning	Mildly Impaired	No change		Moderately Impaired	Mildly Impaired
Depression & Anxiety	Severe	Moderate		Severe	Mild

☐ Option 1 ☐ Option 2

Would you change your choice if the number of patients affected was different?

☐ Yes
☐ No

Question 2:

Which of the following would you prefer?

	Option 1 Current	Option 1 Gain		Option 2 Current	Option 2 Gain
Life Expectancy	Less than 1 year	1 to 5 years		Less than 1 year	No change
Physical Functioning	Severely Impaired	Moderately Impaired		Mildly Impaired	No change
Pain & Discomfort	Extreme	No change	Or	Extreme	Moderate
Cognitive Functioning	Moderately Impaired	No change		Moderately Impaired	Mildly Impaired
Depression & Anxiety	Severe	Moderate		Moderate	No change

☐ Option 1 ☐ Option 2

Would you change your choice if the number of patients affected was different?

☐ Yes
☐ No

Question 3:

Which of the following would you prefer?

	Option 1 Current	Option 1 Gain		Option 2 Current	Option 2 Gain
Life Expectancy	Less than 1 year	1 to 5 years		1 to 5 years	No change
Physical Functioning	Moderately Impaired	No change		Moderately Impaired	Mildly Impaired
Pain & Discomfort	Moderate	No change	Or	Mild	No change
Cognitive Functioning	Severely Impaired	No change		Severely Impaired	Moderately Impaired
Depression & Anxiety	Moderate	Mild		Severe	No change

☐ Option 1 ☐ Option 2

Would you change your choice if the number of patients affected was different?

☐ Yes
☐ No

Question 4:

Which of the following would you prefer?

	Option 1 Current	Option 1 Gain		Option 2 Current	Option 2 Gain
Life Expectancy	1 to 5 years	No change		Less than 1 year	5 years or more
Physical Functioning	Mildly Impaired	No change		Severely Impaired	No change
Pain & Discomfort	Moderate	Mild	Or	Mild	No change
Cognitive Functioning	Mildly Impaired	No change		Severely Impaired	Mildly Impaired
Depression & Anxiety	Severe	Moderate		Mild	No change

☐ Option 1 ☐ Option 2

Would you change your choice if the number of patients affected was different?

☐ Yes
☐ No

Question 5:

Which of the following would you prefer?

	Option 1 Current	Option 1 Gain		Option 2 Current	Option 2 Gain
Life Expectancy	5 years or more	No change		1 to 5 years	5 years or more
Physical Functioning	Severely Impaired	Moderately Impaired		Mildly Impaired	No change
Pain & Discomfort	Moderate	Mild	Or	Extreme	Moderate
Cognitive Functioning	Severely Impaired	No change		Moderately Impaired	Mildly Impaired
Depression & Anxiety	Moderate	No change		Severe	Moderate

☐ Option 1 ☐ Option 2

Would you change your choice if the number of patients affected was different?

☐ Yes
☐ No

Question 6:

Which of the following would you prefer?

	Option 1 Current	Option 1 Gain		Option 2 Current	Option 2 Gain
Life Expectancy	Less than 1 year	No change		5 years or more	No change
Physical Functioning	Moderately Impaired	Mildly Impaired		Mildly Impaired	No change
Pain & Discomfort	Extreme	Moderate	Or	Moderate	Mild
Cognitive Functioning	Mildly Impaired	No change		Severely Impaired	No change
Depression & Anxiety	Moderate	No change		Severe	Moderate

☐ Option 1 ☐ Option 2

Would you change your choice if the number of patients affected was different?

☐ Yes
☐ No

Question 7:

Which of the following would you prefer?

	Current	Gain		Current	Gain
Life Expectancy	1 to 5 years	No change		5 years or more	No change
Physical Functioning	Moderately Impaired	No change		Severely Impaired	Moderately Impaired
Pain & Discomfort	Extreme	No change	Or	Moderate	No change
Cognitive Functioning	Moderately Impaired	Mildly Impaired		Mildly Impaired	No change
Depression & Anxiety	Moderate	Mild		Mild	No change

☐ Option 1 ☐ Option 2

Would you change your choice if the number of patients affected was different?

☐ Yes
☐ No

Question 8:

Which of the following would you prefer?

	Current	Gain		Current	Gain
Life Expectancy	Less than 1 year	1 to 5 years		Less than 1 year	5 years or more
Physical Functioning	Severely Impaired	Mildly Impaired		Severely Impaired	No change
Pain & Discomfort	Mild	No change	Or	Extreme	No change
Cognitive Functioning	Severely Impaired	Moderately Impaired		Severely Impaired	No change
Depression & Anxiety	Moderate	Mild		Severe	No change

☐ Option 1 ☐ Option 2

Would you change your choice if the number of patients affected was different?

☐ Yes
☐ No

Question 9:

Which of the following would you prefer?

	Current	Gain		Current	Gain
Life Expectancy	Less than 1 year	5 years or more		Less than 1 year	1 to 5 years
Physical Functioning	Severely Impaired	Mildly Impaired		Mildly Impaired	No change
Pain & Discomfort	Extreme	Mild	Or	Moderate	Mild
Cognitive Functioning	Mildly Impaired	No change		Moderately Impaired	Mildly Impaired
Depression & Anxiety	Moderate	Mild		Severe	Mild

☐ Option 1 ☐ Option 2

Would you change your choice if the number of patients affected was different?

☐ Yes
☐ No

Question 10:

Which of the following would you prefer?

	Current	Gain		Current	Gain
Life Expectancy	Less than 1 year	No change		Less than 1 year	5 years or more
Physical Functioning	Moderately Impaired	No change		Mildly Impaired	No change
Pain & Discomfort	Extreme	No change	Or	Extreme	Mild
Cognitive Functioning	Moderately Impaired	No change		Mildly Impaired	No change
Depression & Anxiety	Moderate	Mild		Severe	Mild

☐ Option 1 ☐ Option 2

Would you change your choice if the number of patients affected was different?

☐ Yes
☐ No

Question 11:

Which of the following would you prefer?

	Current	Gain		Current	Gain
Life Expectancy	Less than 1 year	1 to 5 years		5 years or more	No change
Physical Functioning	Moderately Impaired	Mildly Impaired		Severely Impaired	Mildly Impaired
Pain & Discomfort	Moderate	No change	Or	Extreme	Moderate
Cognitive Functioning	Severely Impaired	No change		Moderately Impaired	No change
Depression & Anxiety	Moderate	Mild		Severe	Moderate

☐ Option 1 ☐ Option 2

Would you change your choice if the number of patients affected was different?

☐ Yes
☐ No

Question 12:

Which of the following would you prefer?

	Current	Gain		Current	Gain
Life Expectancy	Less than 1 year	No change		1 to 5 years	No change
Physical Functioning	Moderately Impaired	No change		Severely Impaired	No change
Pain & Discomfort	Extreme	Moderate	Or	Extreme	No change
Cognitive Functioning	Severely Impaired	Moderately Impaired		Moderately Impaired	Mildly Impaired
Depression & Anxiety	Moderate	No change		Mild	No change

☐ Option 1 ☐ Option 2

Would you change your choice if the number of patients affected was different?

☐ Yes
☐ No

Question 13:

Which of the following would you prefer?

	Current	Gain		Current	Gain
Life Expectancy	1 to 5 years	5 years or more		1 to 5 years	No change
Physical Functioning	Severely Impaired	Moderately Impaired		Mildly Impaired	No change
Pain & Discomfort	Extreme	No change	Or	Extreme	Mild
Cognitive Functioning	Moderately Impaired	No change		Severely Impaired	No change
Depression & Anxiety	Severe	Mild		Moderate	No change

☐ Option 1 ☐ Option 2

Would you change your choice if the number of patients affected was different?

☐ Yes
☐ No

13

Question 14:

Which of the following would you prefer?

	Current	Gain		Current	Gain
Life Expectancy	Less than 1 year	1 to 5 years		Less than 1 year	5 years or more
Physical Functioning	Mildly Impaired	No change		Moderately Impaired	No change
Pain & Discomfort	Extreme	Mild	Or	Extreme	No change
Cognitive Functioning	Moderately Impaired	No change		Mildly Impaired	No change
Depression & Anxiety	Moderate	No change		Moderate	Mild

☐ Option 1 ☐ Option 2

Would you change your choice if the number of patients affected was different?

☐ Yes
☐ No

14

Question 15:

Which of the following would you prefer?

	Current	Gain		Current	Gain
Life Expectancy	Less than 1 year	No change		Less than 1 year	1 to 5 years
Physical Functioning	Severely Impaired	No change		Mildly Impaired	No change
Pain & Discomfort	Moderate	Mild	Or	Mild	No change
Cognitive Functioning	Severely Impaired	Mildly Impaired		Moderately Impaired	No change
Depression & Anxiety	Severe	Mild		Moderate	No change

☐ Option 1 ☐ Option 2

Would you change your choice if the number of patients affected was different?

☐ Yes
☐ No

15

Question 16:

Which of the following would you prefer?

	Current	Gain		Current	Gain
Life Expectancy	Less than 1 year	5 years or more		1 to 5 years	5 years or more
Physical Functioning	Mildly Impaired	No change		Severely Impaired	Mildly Impaired
Pain & Discomfort	Moderate	No change	Or	Mild	No change
Cognitive Functioning	Moderately Impaired	No change		Severely Impaired	No change
Depression & Anxiety	Severe	No change		Severe	Moderate

☐ Option 1 ☐ Option 2

Would you change your choice if the number of patients affected was different?

☐ Yes
☐ No

16

Question 17:

Which of the following would you prefer?

	Current	Gain		Current	Gain
Life Expectancy	1 to 5 years	No change		Less than 1 year	5 years or more
Physical Functioning	Mildly Impaired	No change		Moderately Impaired	No change
Pain & Discomfort	Moderate	Mild	Or	Moderate	No change
Cognitive Functioning	Severely Impaired	Moderately Impaired		Severely Impaired	No change
Depression & Anxiety	Mild	No change		Severe	Moderate

☐ Option 1 ☐ Option 2

Would you change your choice if the number of patients affected was different?

☐ Yes
☐ No

17

Question 18:

Which of the following would you prefer?

	Current	Gain		Current	Gain
Life Expectancy	Less than 1 year	No change		Less than 1 year	5 years or more
Physical Functioning	Moderately Impaired	No change		Severely Impaired	Mildly Impaired
Pain & Discomfort	Extreme	Mild	Or	Extreme	Moderate
Cognitive Functioning	Severely Impaired	No change		Moderately Impaired	No change
Depression & Anxiety	Severe	Mild		Moderate	Mild

☐ Option 1 ☐ Option 2

Would you change your choice if the number of patients affected was different?

☐ Yes
☐ No

18

Question 19:

Which of the following would you prefer?

	Current	Gain		Current	Gain
Life Expectancy	1 to 5 years	No change		1 to 5 years	5 years or more
Physical Functioning	Severely Impaired	Mildly Impaired		Moderately Impaired	Mildly Impaired
Pain & Discomfort	Mild	No change	Or	Extreme	No change
Cognitive Functioning	Moderately Impaired	No change		Severely Impaired	No change
Depression & Anxiety	Mild	No change		Moderate	No change
	☐ Option 1			☐ Option 2	

Would you change your choice if the number of patients affected was different?

☐ Yes
☐ No

Question 20:

Which of the following would you prefer?

	Current	Gain		Current	Gain
Life Expectancy	1 to 5 years	5 years or more		5 years or more	No change
Physical Functioning	Severely Impaired	Mildly Impaired		Mildly Impaired	No change
Pain & Discomfort	Extreme	Mild	Or	Extreme	No change
Cognitive Functioning	Mildly Impaired	No change		Severely Impaired	Moderately Impaired
Depression & Anxiety	Severe	Mild		Moderate	Mild
	☐ Option 1			☐ Option 2	

Would you change your choice if the number of patients affected was different?

☐ Yes
☐ No

Question 21:

Which of the following would you prefer?

	Current	Gain		Current	Gain
Life Expectancy	1 to 5 years	5 years or more		Less than 1 year	No change
Physical Functioning	Moderately Impaired	No change		Severely Impaired	Moderately Impaired
Pain & Discomfort	Extreme	Mild	Or	Moderate	No change
Cognitive Functioning	Severely Impaired	No change		Mildly Impaired	No change
Depression & Anxiety	Moderate	No change		Moderate	Mild
	☐ Option 1			☐ Option 2	

Would you change your choice if the number of patients affected was different?

☐ Yes
☐ No

Question 22:

Which of the following would you prefer?

	Current	Gain		Current	Gain
Life Expectancy	Less than 1 year	No change		1 to 5 years	5 years or more
Physical Functioning	Severely Impaired	No change		Mildly Impaired	No change
Pain & Discomfort	Extreme	Mild	Or	Moderate	No change
Cognitive Functioning	Severely Impaired	Moderately Impaired		Moderately Impaired	No change
Depression & Anxiety	Mild	No change		Severe	Mild
	☐ Option 1			☐ Option 2	

Would you change your choice if the number of patients affected was different?

☐ Yes
☐ No

Question 23:

Which of the following would you prefer?

	Current	Gain		Current	Gain
Life Expectancy	1 to 5 years	No change		Less than 1 year	1 to 5 years
Physical Functioning	Severely Impaired	Moderately Impaired		Moderately Impaired	Mildly Impaired
Pain & Discomfort	Extreme	Moderate	Or	Extreme	Mild
Cognitive Functioning	Moderately Impaired	Mildly Impaired		Severely Impaired	Mildly Impaired
Depression & Anxiety	Moderate	No change		Severe	No change
	☐ Option 1			☐ Option 2	

Would you change your choice if the number of patients affected was different?

☐ Yes
☐ No

Question 24:

Which of the following would you prefer?

	Current	Gain		Current	Gain
Life Expectancy	Less than 1 year	No change		Less than 1 year	5 years or more
Physical Functioning	Moderately Impaired	No change		Severely Impaired	Moderately Impaired
Pain & Discomfort	Moderate	No change	Or	Extreme	Moderate
Cognitive Functioning	Severely Impaired	No change		Moderately Impaired	Mildly Impaired
Depression & Anxiety	Severe	No change		Moderate	Mild
	☐ Option 1			☐ Option 2	

Would you change your choice if the number of patients affected was different?

☐ Yes
☐ No

Question 25:

Which of the following would you prefer?

	Option 1: Current	Option 1: Gain		Option 2: Current	Option 2: Gain
Life Expectancy	Less than 1 year	5 years or more		Less than 1 year	1 to 5 years
Physical Functioning	Moderately Impaired	Mildly Impaired		Severely Impaired	No change
Pain & Discomfort	Moderate	Mild	Or	Extreme	Moderate
Cognitive Functioning	Severely Impaired	Mildly Impaired		Severely Impaired	Moderately Impaired
Depression & Anxiety	Mild	No change		Severe	Mild

☐ Option 1   ☐ Option 2

Would you change your choice if the number of patients affected was different?

☐ Yes
☐ No

Question 26:

Which of the following would you prefer?

	Option 1: Current	Option 1: Gain		Option 2: Current	Option 2: Gain
Life Expectancy	5 years or more	No change		1 to 5 years	No change
Physical Functioning	Severely Impaired	Mildly Impaired		Moderately Impaired	Mildly Impaired
Pain & Discomfort	Extreme	No change	Or	Extreme	Moderate
Cognitive Functioning	Moderately Impaired	Mildly Impaired		Severely Impaired	No change
Depression & Anxiety	Mild	No change		Severe	Mild

☐ Option 1   ☐ Option 2

Would you change your choice if the number of patients affected was different?

☐ Yes
☐ No

Question 27:

Which of the following would you prefer?

	Option 1: Current	Option 1: Gain		Option 2: Current	Option 2: Gain
Life Expectancy	5 years or more	No change		Less than 1 year	No change
Physical Functioning	Severely Impaired	No change		Moderately Impaired	Mildly Impaired
Pain & Discomfort	Mild	No change	Or	Moderate	Mild
Cognitive Functioning	Severely Impaired	No change		Severely Impaired	Moderately Impaired
Depression & Anxiety	Severe	No change		Moderate	No change

☐ Option 1   ☐ Option 2

Would you change your choice if the number of patients affected was different?

☐ Yes
☐ No

Question 28:

Which of the following would you prefer?

	Option 1: Current	Option 1: Gain		Option 2: Current	Option 2: Gain
Life Expectancy	Less than 1 year	No change		1 to 5 years	No change
Physical Functioning	Moderately Impaired	Mildly Impaired		Moderately Impaired	No change
Pain & Discomfort	Mild	No change	Or	Moderate	No change
Cognitive Functioning	Severely Impaired	Mildly Impaired		Moderately Impaired	Mildly Impaired
Depression & Anxiety	Severe	Moderate		Mild	No change

☐ Option 1   ☐ Option 2

Would you change your choice if the number of patients affected was different?

☐ Yes
☐ No

Question 29:

Which of the following would you prefer?

	Option 1: Current	Option 1: Gain		Option 2: Current	Option 2: Gain
Life Expectancy	Less than 1 year	No change		Less than 1 year	5 years or more
Physical Functioning	Moderately Impaired	No change		Moderately Impaired	Mildly Impaired
Pain & Discomfort	Moderate	No change	Or	Mild	No change
Cognitive Functioning	Mildly Impaired	No change		Severely Impaired	No change
Depression & Anxiety	Mild	No change		Moderate	Mild

☐ Option 1   ☐ Option 2

Would you change your choice if the number of patients affected was different?

☐ Yes
☐ No

Question 30:

Which of the following would you prefer?

	Option 1: Current	Option 1: Gain		Option 2: Current	Option 2: Gain
Life Expectancy	Less than 1 year	No change		Less than 1 year	5 years or more
Physical Functioning	Moderately Impaired	No change		Mildly Impaired	No change
Pain & Discomfort	Extreme	No change	Or	Extreme	Mild
Cognitive Functioning	Moderately Impaired	No change		Mildly Impaired	No change
Depression & Anxiety	Moderate	Mild		Severe	Mild

☐ Option 1   ☐ Option 2

Would you change your choice if the number of patients affected was different?

☐ Yes
☐ No

Group 3

Question 1:

Which of the following would you prefer?

	Option 1 Current	Option 1 Gain		Option 2 Current	Option 2 Gain
Life Expectancy	Less than 1 year	1 to 5 years		Less than 1 year	No change
Physical Functioning	Severely Impaired	No change		Moderately Impaired	No change
Pain & Discomfort	Moderate	Mild	Or	Mild	No change
Cognitive Functioning	Mildly Impaired	No change		Moderately Impaired	Mildly Impaired
Depression & Anxiety	Severe	Moderate		Severe	Mild

☐ Option 1 ☐ Option 2

Would you change your choice if the number of patients affected was different?

☐ Yes
☐ No

Question 2:

Which of the following would you prefer?

	Option 1 Current	Option 1 Gain		Option 2 Current	Option 2 Gain
Life Expectancy	1 to 5 years	5 years or more		Less than 1 year	1 to 5 years
Physical Functioning	Severely Impaired	Moderately Impaired		Moderately Impaired	No change
Pain & Discomfort	Moderate	No change	Or	Moderate	Mild
Cognitive Functioning	Severely Impaired	Moderately Impaired		Mildly Impaired	No Change
Depression & Anxiety	Severe	Mild		Severe	Moderate

☐ Option 1 ☐ Option 2

Would you change your choice if the number of patients affected was different?

☐ Yes
☐ No

Question 3:

Which of the following would you prefer?

	Option 1 Current	Option 1 Gain		Option 2 Current	Option 2 Gain
Life Expectancy	Less than 1 year	1 to 5 years		Less than 1 year	No change
Physical Functioning	Moderately Impaired	Mildly Impaired		Mildly Impaired	No change
Pain & Discomfort	Extreme	Moderate	Or	Mild	No change
Cognitive Functioning	Severely Impaired	Moderately Impaired		Moderately Impaired	No change
Depression & Anxiety	Moderate	Mild		Severe	No change

☐ Option 1 ☐ Option 2

Would you change your choice if the number of patients affected was different?

☐ Yes
☐ No

Question 4:

Which of the following would you prefer?

	Option 1 Current	Option 1 Gain		Option 2 Current	Option 2 Gain
Life Expectancy	5 years or more	No change		Less than 1 year	No change
Physical Functioning	Severely Impaired	Moderately Impaired		Severely Impaired	No change
Pain & Discomfort	Mild	No change	Or	Extreme	Moderate
Cognitive Functioning	Moderately Impaired	Mildly Impaired		Moderately Impaired	No change
Depression & Anxiety	Severe	Mild		Severe	Moderate

☐ Option 1 ☐ Option 2

Would you change your choice if the number of patients affected was different?

☐ Yes
☐ No

Question 5:

Which of the following would you prefer?

	Option 1 Current	Option 1 Gain		Option 2 Current	Option 2 Gain
Life Expectancy	5 years or more	No change		Less than 1 year	5 years or more
Physical Functioning	Severely Impaired	Mildly Impaired		Moderately Impaired	No change
Pain & Discomfort	Extreme	No change	Or	Moderate	Mild
Cognitive Functioning	Moderately Impaired	Mildly Impaired		Moderately Impaired	No change
Depression & Anxiety	Severe	Mild		Mild	No change

☐ Option 1 ☐ Option 2

Would you change your choice if the number of patients affected was different?

☐ Yes
☐ No

Question 6:

Which of the following would you prefer?

	Option 1 Current	Option 1 Gain		Option 2 Current	Option 2 Gain
Life Expectancy	Less than 1 year	5 years or more		Less than 1 year	1 to 5 years
Physical Functioning	Moderately Impaired	Mildly Impaired		Severely Impaired	No change
Pain & Discomfort	Extreme	Mild	Or	Moderate	Mild
Cognitive Functioning	Severely Impaired	Moderately Impaired		Severely Impaired	Mildly Impaired
Depression & Anxiety	Severe	Moderate		Moderate	Mild

☐ Option 1 ☐ Option 2

Would you change your choice if the number of patients affected was different?

☐ Yes
☐ No

Question 7:

Which of the following would you prefer?

	Current	Gain		Current	Gain
Life Expectancy	1 to 5 years	5 years or more		Less than 1 year	No change
Physical Functioning	Severely Impaired	Mildly Impaired		Severely Impaired	No change
Pain & Discomfort	Extreme	Moderate	Or	Extreme	Mild
Cognitive Functioning	Severely Impaired	Mildly Impaired		Mildly Impaired	No change
Depression & Anxiety	Severe	No change		Severe	Mild

☐ Option 1 ☐ Option 2

Would you change your choice if the number of patients affected was different?

☐ Yes
☐ No

7

Question 8:

Which of the following would you prefer?

	Current	Gain		Current	Gain
Life Expectancy	1 to 5 years	5 years or more		Less than 1 year	1 to 5 years
Physical Functioning	Moderately Impaired	No change		Severely Impaired	No change
Pain & Discomfort	Extreme	Moderate	Or	Extreme	Mild
Cognitive Functioning	Severely Impaired	No change		Mildly Impaired	No change
Depression & Anxiety	Mild	No change		Moderate	No change

☐ Option 1 ☐ Option 2

Would you change your choice if the number of patients affected was different?

☐ Yes
☐ No

8

Question 9:

Which of the following would you prefer?

	Current	Gain		Current	Gain
Life Expectancy	1 to 5 years	No change		1 to 5 years	5 years or more
Physical Functioning	Severely Impaired	Mildly Impaired		Moderately Impaired	Mildly Impaired
Pain & Discomfort	Extreme	Mild	Or	Extreme	No change
Cognitive Functioning	Moderately Impaired	Mildly Impaired		Mildly Impaired	No change
Depression & Anxiety	Severe	Moderate		Severe	No change

☐ Option 1 ☐ Option 2

Would you change your choice if the number of patients affected was different?

☐ Yes
☐ No

9

Question 10:

Which of the following would you prefer?

	Current	Gain		Current	Gain
Life Expectancy	Less than 1 year	No change		Less than 1 year	5 years or more
Physical Functioning	Moderately Impaired	No change		Mildly Impaired	No change
Pain & Discomfort	Extreme	No change	Or	Extreme	Mild
Cognitive Functioning	Moderately Impaired	No change		Mildly Impaired	No change
Depression & Anxiety	Moderate	Mild		Severe	Mild

☐ Option 1 ☐ Option 2

Would you change your choice if the number of patients affected was different?

☐ Yes
☐ No

10

Question 11:

Which of the following would you prefer?

	Current	Gain		Current	Gain
Life Expectancy	5 years or more	No change		1 to 5 years	No change
Physical Functioning	Severely Impaired	No change		Severely Impaired	Mildly Impaired
Pain & Discomfort	Extreme	Moderate	Or	Extreme	No change
Cognitive Functioning	Moderately Impaired	Mildly Impaired		Severely Impaired	Moderately Impaired
Depression & Anxiety	Moderate	No change		Mild	No change

☐ Option 1 ☐ Option 2

Would you change your choice if the number of patients affected was different?

☐ Yes
☐ No

11

Question 12:

Which of the following would you prefer?

	Current	Gain		Current	Gain
Life Expectancy	Less than 1 year	1 to 5 years		1 to 5 years	5 years or more
Physical Functioning	Moderately Impaired	No change		Severely Impaired	Mildly Impaired
Pain & Discomfort	Extreme	Moderate	Or	Moderate	Mild
Cognitive Functioning	Severely Impaired	Mildly Impaired		Severely Impaired	No change
Depression & Anxiety	Mild	No change		Moderate	Mild

☐ Option 1 ☐ Option 2

Would you change your choice if the number of patients affected was different?

☐ Yes
☐ No

12

Question 13:

Which of the following would you prefer?

	Option 1: Current	Option 1: Gain	Or	Option 2: Current	Option 2: Gain
Life Expectancy	Less than 1 year	No change		5 years or more	No change
Physical Functioning	Mildly Impaired	No change		Moderately Impaired	No change
Pain & Discomfort	Moderate	No change	Or	Mild	No change
Cognitive Functioning	Moderately Impaired	Mildly Impaired		Severely Impaired	Moderately Impaired
Depression & Anxiety	Moderate	Mild		Mild	No change

☐ Option 1 ☐ Option 2

Would you change your choice if the number of patients affected was different?

☐ Yes
☐ No

Question 14:

Which of the following would you prefer?

	Option 1: Current	Option 1: Gain	Or	Option 2: Current	Option 2: Gain
Life Expectancy	Less than 1 year	5 years or more		5 years or more	No change
Physical Functioning	Moderately Impaired	No change		Severely Impaired	Moderately Impaired
Pain & Discomfort	Mild	No change	Or	Extreme	Mild
Cognitive Functioning	Moderately Impaired	Mildly Impaired		Severely Impaired	No change
Depression & Anxiety	Severe	No change		Mild	No change

☐ Option 1 ☐ Option 2

Would you change your choice if the number of patients affected was different?

☐ Yes
☐ No

Question 15:

Which of the following would you prefer?

	Option 1: Current	Option 1: Gain	Or	Option 2: Current	Option 2: Gain
Life Expectancy	5 years or more	No change		1 to 5 years	5 years or more
Physical Functioning	Moderately Impaired	Mildly Impaired		Severely Impaired	Moderately Impaired
Pain & Discomfort	Moderate	Mild	Or	Moderate	No change
Cognitive Functioning	Severely Impaired	Mildly Impaired		Mildly Impaired	No change
Depression & Anxiety	Severe	Mild		Severe	No change

☐ Option 1 ☐ Option 2

Would you change your choice if the number of patients affected was different?

☐ Yes
☐ No

Question 16:

Which of the following would you prefer?

	Option 1: Current	Option 1: Gain	Or	Option 2: Current	Option 2: Gain
Life Expectancy	Less than 1 year	No change		1 to 5 years	No change
Physical Functioning	Severely Impaired	No change		Moderately Impaired	Mildly Impaired
Pain & Discomfort	Moderate	Mild	Or	Extreme	Mild
Cognitive Functioning	Mildly Impaired	No change		Moderately Impaired	No change
Depression & Anxiety	Severe	Moderate		Moderate	No change

☐ Option 1 ☐ Option 2

Would you change your choice if the number of patients affected was different?

☐ Yes
☐ No

Question 17:

Which of the following would you prefer?

	Option 1: Current	Option 1: Gain	Or	Option 2: Current	Option 2: Gain
Life Expectancy	Less than 1 year	5 years or more		Less than 1 year	No change
Physical Functioning	Severely Impaired	No change		Mildly Impaired	No change
Pain & Discomfort	Moderate	No change	Or	Extreme	Moderate
Cognitive Functioning	Severely Impaired	Moderately Impaired		Severely Impaired	No change
Depression & Anxiety	Severe	Mild		Severe	No change

☐ Option 1 ☐ Option 2

Would you change your choice if the number of patients affected was different?

☐ Yes
☐ No

Question 18:

Which of the following would you prefer?

	Option 1: Current	Option 1: Gain	Or	Option 2: Current	Option 2: Gain
Life Expectancy	1 to 5 years	5 years or more		1 to 5 years	No change
Physical Functioning	Severely Impaired	Moderately Impaired		Severely Impaired	No change
Pain & Discomfort	Moderate	Mild	Or	Mild	No change
Cognitive Functioning	Moderately Impaired	Mildly Impaired		Moderately Impaired	No change
Depression & Anxiety	Severe	No change		Severe	Moderate

☐ Option 1 ☐ Option 2

Would you change your choice if the number of patients affected was different?

☐ Yes
☐ No

Question 19:

Which of the following would you prefer?

	Option 1: Current	Option 1: Gain		Option 2: Current	Option 2: Gain
Life Expectancy	Less than 1 year	5 years or more		1 to 5 years	5 years or more
Physical Functioning	Severely Impaired	Mildly Impaired		Moderately Impaired	No change
Pain & Discomfort	Extreme	Moderate	Or	Extreme	Mild
Cognitive Functioning	Severely Impaired	No change		Moderately Impaired	No change
Depression & Anxiety	Severe	No change		Mild	No change

☐ Option 1 ☐ Option 2

Would you change your choice if the number of patients affected was different?

☐ Yes
☐ No

Question 20:

Which of the following would you prefer?

	Option 1: Current	Option 1: Gain		Option 2: Current	Option 2: Gain
Life Expectancy	1 to 5 years	5 years or more		5 years or more	No change
Physical Functioning	Severely Impaired	Mildly Impaired		Mildly Impaired	No change
Pain & Discomfort	Extreme	Mild	Or	Extreme	No change
Cognitive Functioning	Mildly Impaired	No change		Severely Impaired	Moderately Impaired
Depression & Anxiety	Severe	Mild		Moderate	Mild

☐ Option 1 ☐ Option 2

Would you change your choice if the number of patients affected was different?

☐ Yes
☐ No

Question 21:

Which of the following would you prefer?

	Option 1: Current	Option 1: Gain		Option 2: Current	Option 2: Gain
Life Expectancy	1 to 5 years	5 years or more		5 years or more	No change
Physical Functioning	Severely Impaired	Moderately Impaired		Moderately Impaired	Mildly Impaired
Pain & Discomfort	Extreme	Moderate	Or	Extreme	No change
Cognitive Functioning	Severely Impaired	Mildly Impaired		Moderately Impaired	Mildly Impaired
Depression & Anxiety	Severe	No change		Severe	Mild

☐ Option 1 ☐ Option 2

Would you change your choice if the number of patients affected was different?

☐ Yes
☐ No

Question 22:

Which of the following would you prefer?

	Option 1: Current	Option 1: Gain		Option 2: Current	Option 2: Gain
Life Expectancy	1 to 5 years	No change		5 years or more	No change
Physical Functioning	Severely Impaired	Mildly Impaired		Severely Impaired	No change
Pain & Discomfort	Moderate	Mild	Or	Extreme	Moderate
Cognitive Functioning	Severely Impaired	Moderately Impaired		Mildly Impaired	No change
Depression & Anxiety	Severe	No change		Mild	No change

☐ Option 1 ☐ Option 2

Would you change your choice if the number of patients affected was different?

☐ Yes
☐ No

Question 23:

Which of the following would you prefer?

	Option 1: Current	Option 1: Gain		Option 2: Current	Option 2: Gain
Life Expectancy	5 years or more	No change		1 to 5 years	5 years or more
Physical Functioning	Mildly Impaired	No change		Moderately Impaired	Mildly Impaired
Pain & Discomfort	Extreme	Mild	Or	Moderate	Mild
Cognitive Functioning	Severely Impaired	Mildly Impaired		Severely Impaired	No change
Depression & Anxiety	Severe	No change		Mild	No change

☐ Option 1 ☐ Option 2

Would you change your choice if the number of patients affected was different?

☐ Yes
☐ No

Question 24:

Which of the following would you prefer?

	Option 1: Current	Option 1: Gain		Option 2: Current	Option 2: Gain
Life Expectancy	1 to 5 years	No change		Less than 1 year	1 to 5 years
Physical Functioning	Severely Impaired	Moderately Impaired		Severely Impaired	Moderately Impaired
Pain & Discomfort	Extreme	No change	Or	Mild	No change
Cognitive Functioning	Mildly Impaired	No change		Moderately Impaired	Mildly Impaired
Depression & Anxiety	Moderate	No change		Severe	Moderate

☐ Option 1 ☐ Option 2

Would you change your choice if the number of patients affected was different?

☐ Yes
☐ No

Question 25:

Which of the following would you prefer?

	Option 1 Current	Option 1 Gain	Or	Option 2 Current	Option 2 Gain
Life Expectancy	1 to 5 years	5 years or more		1 to 5 years	No change
Physical Functioning	Severely Impaired	Mildly Impaired		Severely Impaired	Moderately Impaired
Pain & Discomfort	Moderate	Mild		Moderate	No change
Cognitive Functioning	Mildly Impaired	No change		Severely Impaired	Mildly Impaired
Depression & Anxiety	Mild	No change		Moderate	No change

☐ Option 1 ☐ Option 2

Would you change your choice if the number of patients affected was different?

☐ Yes
☐ No

Question 26:

Which of the following would you prefer?

	Option 1 Current	Option 1 Gain	Or	Option 2 Current	Option 2 Gain
Life Expectancy	5 years or more	No change		Less than 1 year	No change
Physical Functioning	Severely Impaired	No change		Moderately Impaired	Mildly Impaired
Pain & Discomfort	Mild	No change		Extreme	Mild
Cognitive Functioning	Severely Impaired	Moderately Impaired		Moderately Impaired	No change
Depression & Anxiety	Moderate	No change		Severe	Mild

☐ Option 1 ☐ Option 2

Would you change your choice if the number of patients affected was different?

☐ Yes
☐ No

Question 27:

Which of the following would you prefer?

	Option 1 Current	Option 1 Gain	Or	Option 2 Current	Option 2 Gain
Life Expectancy	1 to 5 years	No change		Less than 1 year	No change
Physical Functioning	Severely Impaired	Moderately Impaired		Moderately Impaired	Mildly Impaired
Pain & Discomfort	Extreme	Moderate		Mild	No change
Cognitive Functioning	Severely Impaired	Mildly Impaired		Moderately Impaired	Mildly Impaired
Depression & Anxiety	Severe	Mild		Severe	No change

☐ Option 1 ☐ Option 2

Would you change your choice if the number of patients affected was different?

☐ Yes
☐ No

Question 28:

Which of the following would you prefer?

	Option 1 Current	Option 1 Gain	Or	Option 2 Current	Option 2 Gain
Life Expectancy	Less than 1 year	5 years or more		1 to 5 years	No change
Physical Functioning	Mildly Impaired	No change		Moderately Impaired	No change
Pain & Discomfort	Extreme	Mild		Mild	No change
Cognitive Functioning	Severely Impaired	No change		Mildly Impaired	No change
Depression & Anxiety	Moderate	Mild		Moderate	No change

☐ Option 1 ☐ Option 2

Would you change your choice if the number of patients affected was different?

☐ Yes
☐ No

Question 29:

Which of the following would you prefer?

	Option 1 Current	Option 1 Gain	Or	Option 2 Current	Option 2 Gain
Life Expectancy	1 to 5 years	5 years or more		Less than 1 year	1 to 5 years
Physical Functioning	Severely Impaired	No change		Severely Impaired	Moderately Impaired
Pain & Discomfort	Moderate	Mild		Extreme	Moderate
Cognitive Functioning	Severely Impaired	Moderately Impaired		Severely Impaired	No change
Depression & Anxiety	Severe	Moderate		Mild	No change

☐ Option 1 ☐ Option 2

Would you change your choice if the number of patients affected was different?

☐ Yes
☐ No

Question 30:

Which of the following would you prefer?

	Option 1 Current	Option 1 Gain	Or	Option 2 Current	Option 2 Gain
Life Expectancy	Less than 1 year	No change		Less than 1 year	5 years or more
Physical Functioning	Moderately Impaired	No change		Mildly Impaired	No change
Pain & Discomfort	Extreme	No change		Extreme	Mild
Cognitive Functioning	Moderately Impaired	No change		Mildly Impaired	No change
Depression & Anxiety	Moderate	Mild		Severe	Mild

☐ Option 1 ☐ Option 2

Would you change your choice if the number of patients affected was different?

☐ Yes
☐ No

Exercise 4 ('How do we make choices when gains may seem small?') Questions

Question 1:

There is a condition in which current treatment involves many sessions of radiation. Patients have to come to the treatment centre 5 days a week for 6 consecutive weeks. The cost of treatment to the healthcare system is $8,000 per patient per year.

There is a new type of radiation treatment in which patients only need to go to the centre once. It works as well as the current treatment and the cost is $8,000 per patient.

Which treatment would you choose?

- ☐ Current treatment
- ☐ New treatment

1

Question 1 continued:

Would you choose the new treatment if it costs:

- $9,000 per patient? ☐ Yes ☐ No
- $10,000 per patient? ☐ Yes ☐ No
- $11,000 per patient? ☐ Yes ☐ No
- $12,000 per patient? ☐ Yes ☐ No
- $13,000 per patient? ☐ Yes ☐ No
- $14,000 per patient? ☐ Yes ☐ No
- $15,000 per patient? ☐ Yes ☐ No
- $16,000 per patient? ☐ Yes ☐ No
- $17,000 per patient? ☐ Yes ☐ No
- $18,000 per patient? ☐ Yes ☐ No
- $19,000 per patient? ☐ Yes ☐ No
- $20,000 per patient? ☐ Yes ☐ No

2

Question 2:

There is a condition in which current treatment involves an injection every 4 hours throughout the day and night. The cost of treatment to the healthcare system is $2,000 per patient per year.

There is a new medical device that delivers the same medication automatically through small tubing and a needle inserted into the skin. They need to be replaced every 3 days. The cost of this treatment is $2,000 per patient.

Which treatment would you fund?

- ☐ Current treatment
- ☐ New treatment

3

Question 2 continued:

Would you choose the new treatment if it costs:

- $2,500 per patient? ☐ Yes ☐ No
- $3,000 per patient? ☐ Yes ☐ No
- $3,500 per patient? ☐ Yes ☐ No
- $4,000 per patient? ☐ Yes ☐ No
- $4,500 per patient? ☐ Yes ☐ No
- $5,000 per patient? ☐ Yes ☐ No
- $5,500 per patient? ☐ Yes ☐ No
- $6,000 per patient? ☐ Yes ☐ No
- $6,500 per patient? ☐ Yes ☐ No
- $7,000 per patient? ☐ Yes ☐ No
- $7,500 per patient? ☐ Yes ☐ No
- $8,000 per patient? ☐ Yes ☐ No

4

Question 3:

There is a condition in which current treatment involves an intravenous medication that is given in the hospital over 4 hours every 2 weeks for life. The cost of treatment to the healthcare system is $200,000 per patient per year.

There is a new oral treatment that involves taking 1 pill 3 times per day. The pill is as effective as the IV medication at controlling the condition. The cost of this treatment is $200,000 per patient.

Which treatment would you fund?

- ☐ Current treatment
- ☐ New treatment

5

Question 3 continued:

Would you choose the new treatment if it costs:

- $225,000 per patient? ☐ Yes ☐ No
- $250,000 per patient? ☐ Yes ☐ No
- $275,000 per patient? ☐ Yes ☐ No
- $300,000 per patient? ☐ Yes ☐ No
- $325,000 per patient? ☐ Yes ☐ No
- $350,000 per patient? ☐ Yes ☐ No
- $375,000 per patient? ☐ Yes ☐ No
- $400,000 per patient? ☐ Yes ☐ No
- $425,000 per patient? ☐ Yes ☐ No
- $450,000 per patient? ☐ Yes ☐ No
- $475,000 per patient? ☐ Yes ☐ No
- $500,000 per patient? ☐ Yes ☐ No

6

Question 4:

There is a condition in which current treatment involves taking 10 pills six times per day for life. The cost of treatment to the healthcare system is $12,000 per patient per year.

There is a new oral treatment that involves taking 1 pill 6 times per day. It is as effective as current treatment. The cost of this treatment is $12,000 per patient.

Which treatment would you fund?

- ☐ Current treatment
- ☐ New treatment

7

Question 4 continued:

Would you choose the new treatment if it costs:

- $12,000 per patient? ☐ Yes ☐ No
- $16,000 per patient? ☐ Yes ☐ No
- $20,000 per patient? ☐ Yes ☐ No
- $24,000 per patient? ☐ Yes ☐ No
- $28,000 per patient? ☐ Yes ☐ No
- $32,000 per patient? ☐ Yes ☐ No
- $36,000 per patient? ☐ Yes ☐ No
- $40,000 per patient? ☐ Yes ☐ No
- $44,000 per patient? ☐ Yes ☐ No
- $48,000 per patient? ☐ Yes ☐ No
- $52,000 per patient? ☐ Yes ☐ No
- $56,000 per patient? ☐ Yes ☐ No

8

Question 5:

There is a genetic condition that causes progressive decline in mental abilities, rigid muscles, tremors, and slowed movement. The condition has no cure and is terminal. Treatment involves managing symptoms when they develop.

All individuals with the mutation will eventually develop the disease. There is a new blood test that will you whether or not you have the mutation. It costs $50.

Would you fund the test?

- ☐ Yes
- ☐ No

Question 5 continued:

Would you fund it if it costs:

$100 per patient? ☐ Yes ☐ No	$150 per patient? ☐ Yes ☐ No	$200 per patient? ☐ Yes ☐ No	$250 per patient? ☐ Yes ☐ No
$300 per patient? ☐ Yes ☐ No	$350 per patient? ☐ Yes ☐ No	$400 per patient? ☐ Yes ☐ No	$450 per patient? ☐ Yes ☐ No
$500 per patient? ☐ Yes ☐ No	$1,000 per patient? ☐ Yes ☐ No	$2,000 per patient? ☐ Yes ☐ No	$3,000 per patient? ☐ Yes ☐ No

Question 6:

There is a condition in which current treatment extends life by three months after diagnosis. After recovering from the treatment, which can take up to 3 weeks, patients feel better for about one month before they start to get worse again and eventually die. The cost of the treatment to the healthcare system is $1,000 per patient per treatment cycle.

There is a new oral treatment that extends life by 3 more months than current treatment. The oral treatment has few side effects, and patients experience few symptoms until the last month, during which they rapidly decline. The cost of this treatment is $1,000 per patient.

Which treatment would you fund?

- ☐ Current treatment
- ☐ New treatment

Question 6 continued:

Would you choose the new treatment if it costs:

$2,000 per patient? ☐ Yes ☐ No	$3,,000 per patient? ☐ Yes ☐ No	$4,000 per patient? ☐ Yes ☐ No	$5,000 per patient? ☐ Yes ☐ No
$10,000 per patient? ☐ Yes ☐ No	$15,000 per patient? ☐ Yes ☐ No	$20,000 per patient? ☐ Yes ☐ No	$25,000 per patient? ☐ Yes ☐ No
$30,000 per patient? ☐ Yes ☐ No	$35,000 per patient? ☐ Yes ☐ No	$40,000 per patient? ☐ Yes ☐ No	$45,000 per patient? ☐ Yes ☐ No

Question 7:

There is a condition in which current treatment involves taking 5 pills every day for life. Individuals take the pills to control blood pressure. The pills have minimal side effects. The cost of the treatment to the healthcare system is $500 per patient per year.

There is a new laser treatment that uses a catheter inserted into a vein to burn the nerves that control blood pressure. It works as well as current treatment, and patients no longer need to take pills. This treatment is repeated every 3 years. The cost of this treatment is $500 per patient.

Which treatment would you fund?

- ☐ Current treatment
- ☐ New treatment

Question 7 continued:

Would you choose the new treatment if it costs:

$1,000 per patient? ☐ Yes ☐ No	$1,,500 per patient? ☐ Yes ☐ No	$2,000 per patient? ☐ Yes ☐ No	$2,500 per patient? ☐ Yes ☐ No
$3,000 per patient? ☐ Yes ☐ No	$3,500 per patient? ☐ Yes ☐ No	$4,000 per patient? ☐ Yes ☐ No	$4,500 per patient? ☐ Yes ☐ No
$5,000 per patient? ☐ Yes ☐ No	$7,000 per patient? ☐ Yes ☐ No	$9,000 per patient? ☐ Yes ☐ No	$12,000 per patient? ☐ Yes ☐ No

Question 8:

Current treatment for certain solid tumours that don't respond to chemotherapy or radiation is surgery. Surgery extends life by 6 months. The cost per patient is $20,000.

There is an innovative treatment in which healthy genes are put into viruses that infect cancer cells and turn off the gene that is causing the cells to grow out of control. The viruses are injected into the tumour. The treatment is effective at slowing growth for about 4 months until the patient's own immune system develops antibodies to the virus, destroying it from delivering the healthy genes to the cancer cells. Survival after the viruses are destroyed is 2 months. The cost per patient is $20,000.

Which treatment would you fund?

- ☐ Current treatment
- ☐ New treatment

Question 8 continued:

Would you choose the new treatment if it costs:

$25,000 per patient? ☐ Yes ☐ No	$30,000 per patient? ☐ Yes ☐ No	$35,000 per patient? ☐ Yes ☐ No	$40,000 per patient? ☐ Yes ☐ No
$45,000 per patient? ☐ Yes ☐ No	$50,000 per patient? ☐ Yes ☐ No	$60,000 per patient? ☐ Yes ☐ No	$70,000 per patient? ☐ Yes ☐ No
$80,000 per patient? ☐ Yes ☐ No	$90,000 per patient? ☐ Yes ☐ No	$95,000 per patient? ☐ Yes ☐ No	$100,000 per patient? ☐ Yes ☐ No
