# Supplementary material for: Moving beyond the Court of Public Opinion: A Citizens’ Jury Exploring the Public’s Values around Funding Decisions for Ultra-Orphan Drugs
Source: Int J Environ Res Public Health. 2022 Dec 30;20(1):633. doi: 10.3390/ijerph20010633 (PMC9819519; doi:10.3390/ijerph20010633)
Supplement: Supplementary file 1 [file ijerph-20-00633-s001.zip › Supplementary Materials S4.pdf]

Supplementary Materials S4

Table S1. Consolidated criteria for reporting qualitative studies (COREQ)		
**No.**	**Item**	**Response**
**Domain 1: research team and reflexivity**		
Personal characteristics		
1.	Which author/s conducted the Citizens' Jury?	AY, DM, JS, and TS facilitated the exercises conducted in the Citizens' Jury (page 4*).
2.	What were the researcher's credentials?	AY holds a BSc in biological sciences and an MSc in health policy research.<br>DM holds a PhD in physics and a Masters of Health Services Administration.<br>JS holds a PhD in biochemistry and a Grad. Dip in Primary Health Care, Public Health.<br>TS holds an MSc in epidemiology and a PhD in public health sciences.
3.	What was their occupation at the time of the study?	AY is a Research Associate at the Health Technology and Policy Unit.<br>DM is a professor in the School of Public Health, University of Alberta.<br>JS is a professor in the School of Public Health, University of Adelaide.<br>TS is the Director of the Health Technology and Policy Unit in the School of Public Health, University of Alberta.
4.	Was the researcher male or female?	AY, JS, and TS are female. DM is male (page 4).
5.	What experience or training did the researcher have?	AY, DM, JS, and TS all have prior experience in facilitating deliberative exercises (page 4).
Relationship with participants		
6.	Was a relationship established prior to study commencement?	No relationship was established until study recruitment began (page 3).
7.	What did the participants know about the researcher?	The participants were familiar with the interviewers' research interests and reasons for doing the research (page 4).
8.	What characteristics were reported about the interviewer/facilitator?	The researchers background and potential biases were reported (page 4).
**Domain 2: study design**		
Theoretical framework		
9.	What methodological orientation was stated to underpin the study?	Pragmatic qualitative research (page 3).
Participant selection		
10.	How were participants selected?	Through purposive and stratified random sampling (page 3-4).
11.	How were participants approached?	Letter mail (page 3-4).
12.	How many participants were in the study?	Sixteen (page 10).
13.	How many people refused to participate or dropped out? Reasons?	None (page 10).
Setting		
14.	Where was the data collected?	Hotel conference centre in Edmonton, Alberta (page 4-5).

Table S1. Consolidated criteria for reporting qualitative studies (COREQ)		
**No.**	**Item**	**Response**
15.	Was anyone else present besides the participants and researchers?	No (page 4-5).
16.	What are the important characteristics of the sample?	Sociodemographic profile matching that of Alberta on the following characteristics: gender, age, education, house income, employment status, ethnicity (page 10).
Data collection		
17.	Were questions, prompts, guides provided by the authors? Was it pilot tested?	Yes. Similar trade-off questions were trialed in previously conducted Citizens' Juries.
18.	Were repeat interviews carried out? If yes, how many?	Not applicable.
19.	Did the research use audio or visual recording to collect the data?	Yes (page 9).
20.	Were field notes made during and/or after the Citizens' Jury?	Yes (page 9).
21.	What was the duration of the Citizens' Jury?	Two and a half days (page 4).
22.	Was data saturation discussed?	Yes.
23.	Were transcripts returned to participants for comment and/or correction?	No.
**Domain 3: analysis and findings**		
Data analysis		
24.	How many data coders coded the data?	Two (page 9).
25.	Did authors provide a description of the coding tree?	Yes (Appendix C).
26.	Were themes identified in advance or derived from the data?	Both (page 9).
27.	What software, if applicable, was used to manage the data?	Not applicable.
28.	Did participants provide feedback on the findings?	Feedback was obtained through 'report back' sessions during the Jury proceedings and by the facilitators who reviewed their notes with the Jurors after each Exercise (page 8).
Reporting		
29.	Were participant quotations presented to illustrate the themes / findings? Was each quotation identified?	Yes (pages 11 – 18).
30.	Was there consistency between the data presented and the findings?	Yes (pages 9 – 19).
31.	Were major themes clearly presented in the findings?	Yes (pages 9 – 19).
32.	Is there a description of diverse cases or discussion of minor themes?	Yes (pages 9 – 19).

*Note: page # indicates on which page of the manuscript the corresponding item information can be found.
