# Supplementary figures and images for: Moving beyond the Court of Public Opinion: A Citizens’ Jury Exploring the Public’s Values around Funding Decisions for Ultra-Orphan Drugs
Source: Int J Environ Res Public Health. 2022 Dec 30;20(1):633. doi: 10.3390/ijerph20010633 (PMC9819519; doi:10.3390/ijerph20010633)

**Supplementary Materials S3**  
Sample coding tree

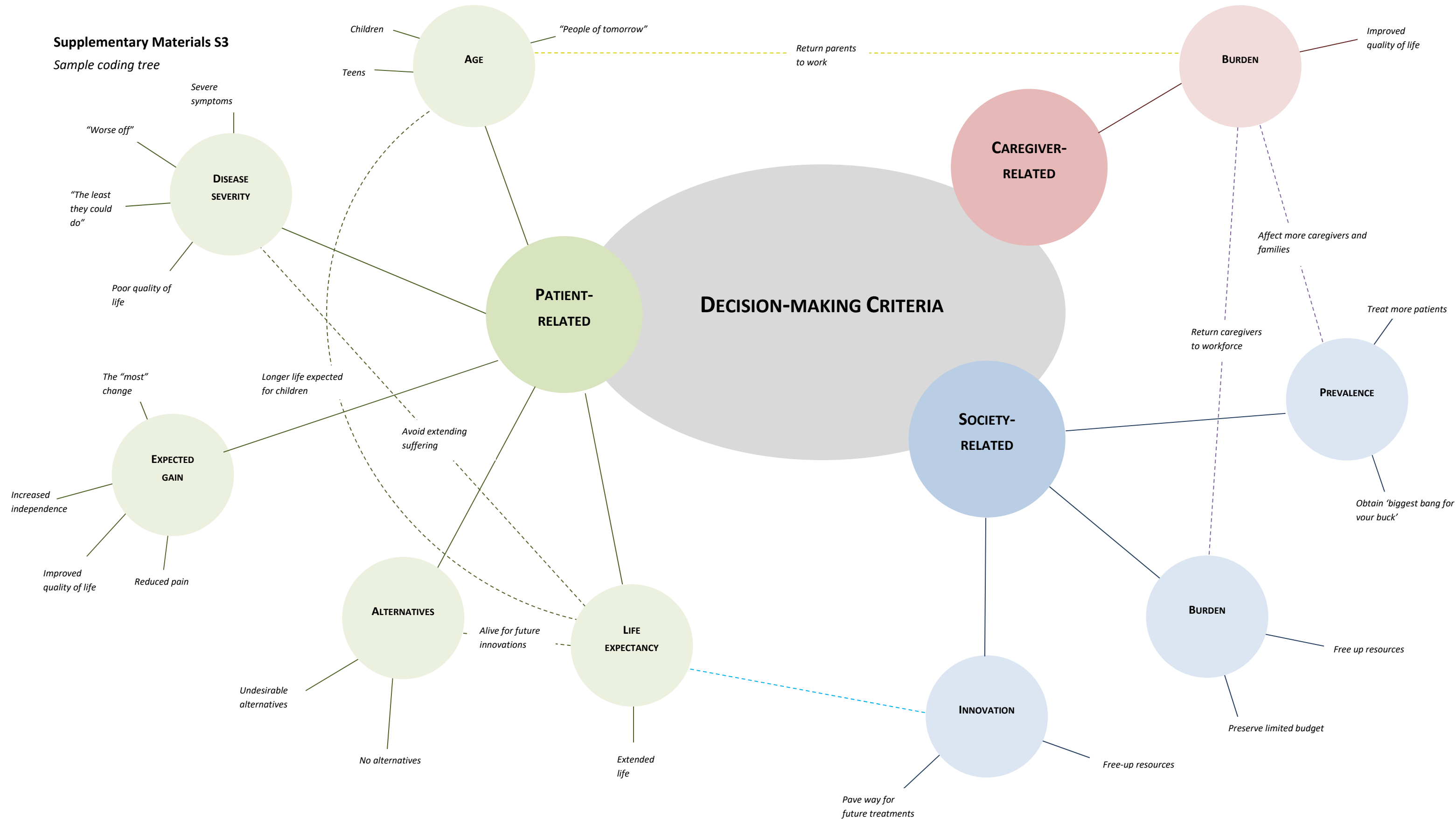

Supplement: Supplementary file 1 [file ijerph-20-00633-s001.zip › Supplementary Materials S3.pdf]
